# Supplementary material for: Chaperone biomarkers of lifespan and penetrance track the dosages of many other proteins
Source: Nat Commun. 2019 Dec 16;10:5725. doi: 10.1038/s41467-019-13664-7 (PMC6914778; doi:10.1038/s41467-019-13664-7)
Supplement: Supplementary file 1 — Supplementary Information [file 41467_2019_13664_MOESM1_ESM.pdf]

Supplementary Information

for

Chaperone biomarkers of lifespan and penetrance track the dosages of many other proteins

by

Burnaevskiy et al.

Table of Contents

Supplementary Figures 1-12..Pgs 2-15

Supplementary Note 1: Defining Pathway Output and Expression Capacity…Pgs. 16-17

Supplementary Note 2: Extracting Pathway and Expression Capacity Information from the Data Variance and Covariance……………………………………………..Pgs. 17-18

Supplementary Note 3: Calculating Variance and Covariance of *G*, *γ*, *L*, and *λ*……………………………………………………………………………Pgs. 18-21

Supplementary Note 4: Two Color Variants Driven by the Same Promoter and Intrinsic Noise of Gene Expression…………………………………………………Pgs. 21-23

Supplementary Note 5: Two Color Variants Driven by Different Promoters and Pathway Variation……………………………………………………………………..…Pgs. 23-25

Supplementary Note 6: Additional Correlations between Phenotypes and Reporter Genes……………………………………………………………………………Pgs. 26-32

Supplementary Note 7: Persistence of Physiological States…………………...Pgs. 33-36

Supplementary Note 8: Trade-offs (including Supplementary Fig. 18)………..Pgs. 37-39

Supplementary Table 4……………………………………………………………….Pg. 40

Supplementary Discussion……………………………………………………Pgs. 41-44

Supplementary References…………………………………………………..Pgs. 45-46

Supplementary Figure 1. Generation of animals to measure intrinsic noise. Cartoon shows how we generated animals for Type I experiments to examine intrinsic noise. Top section shows two homozygous animals being bred to create an F1 hybrid animal. Each reporter gene has its own stereotyped expression pattern for expression levels along the longitudinal axis of the intestine. Here we are showing the pattern for *hsp-16.2* reporters. Bottom panels show what animals with difference degrees of *γ* would look like. Left panel shows that animals with low *γ* would have intestine cells with balanced biallelic expression – in those cells, the merge of the mCherry and mEGFP reporter alleles would appear as yellow. The center panel shows that all intestine cells in animals with cell nonautonomous differences in *γ* would appear shifted toward one or the other allele – that is red-shifted or green-shifted. The right panel shows that exhibit large cell autonomous differences in *γ* would manifest as a randomly arranged array of red, green and yellow (biallelilc) cells.

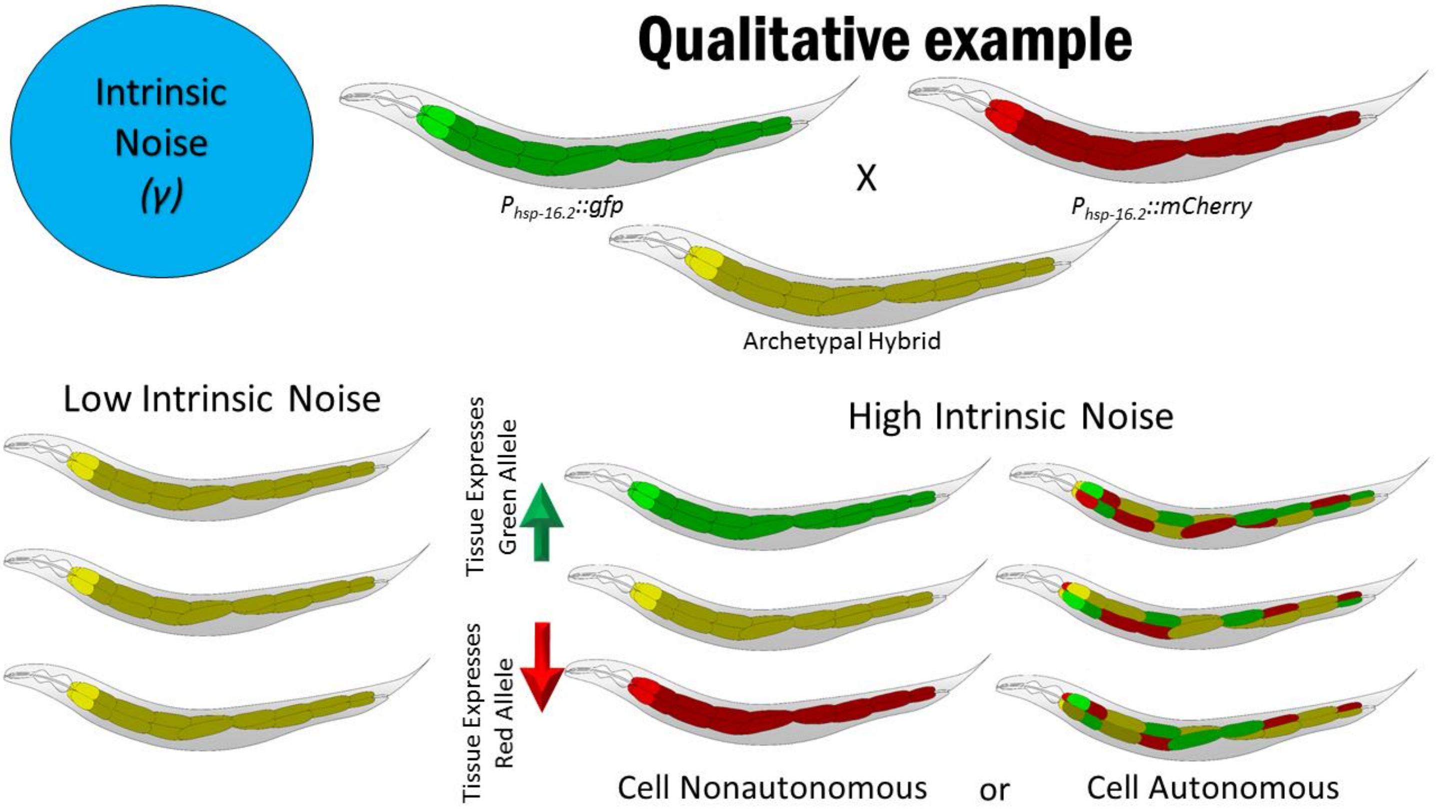

Supplementary Figure 2. Generation of animals to quantify signaling noise. Cartoon shows how we generated animals for the Type II experiments in which we measured signaling noise. Top shows two homozygous animals crossed to create an archetypal hybrid. As in Supplementary Fig. 1, the archetypical hybrid has a stereotyped expression pattern, but this time it is from distinct reporter genes. The combination of any two patterns examined at cell resolution results in different, cell specific slopes (i.e., a ratiometric setpoint) for each pair of genes when many individual cells' expression levels for those two distinct reporters are displayed on a scatter plot. Bottom panels show (left) what animals with small differences in *P* would look like, (center) what animals with large cell nonautonomous differences in *P* would look like, and (right) what animals with large cell nonautonomous differences in *P* would look like. All of these cartoon images are assuming intrinsic noise is minimal; we use this assumption in these figures, because large amounts of intrinsic noise would occlude our ability to detect signaling noise in the two-signal measurement scheme employed in these studies. Cell autonomous signaling noise would be experimentally indistinguishable from intrinsic noise; this requires us to establish some measures of intrinsic noise before attempting to precisely quantify signaling noise.

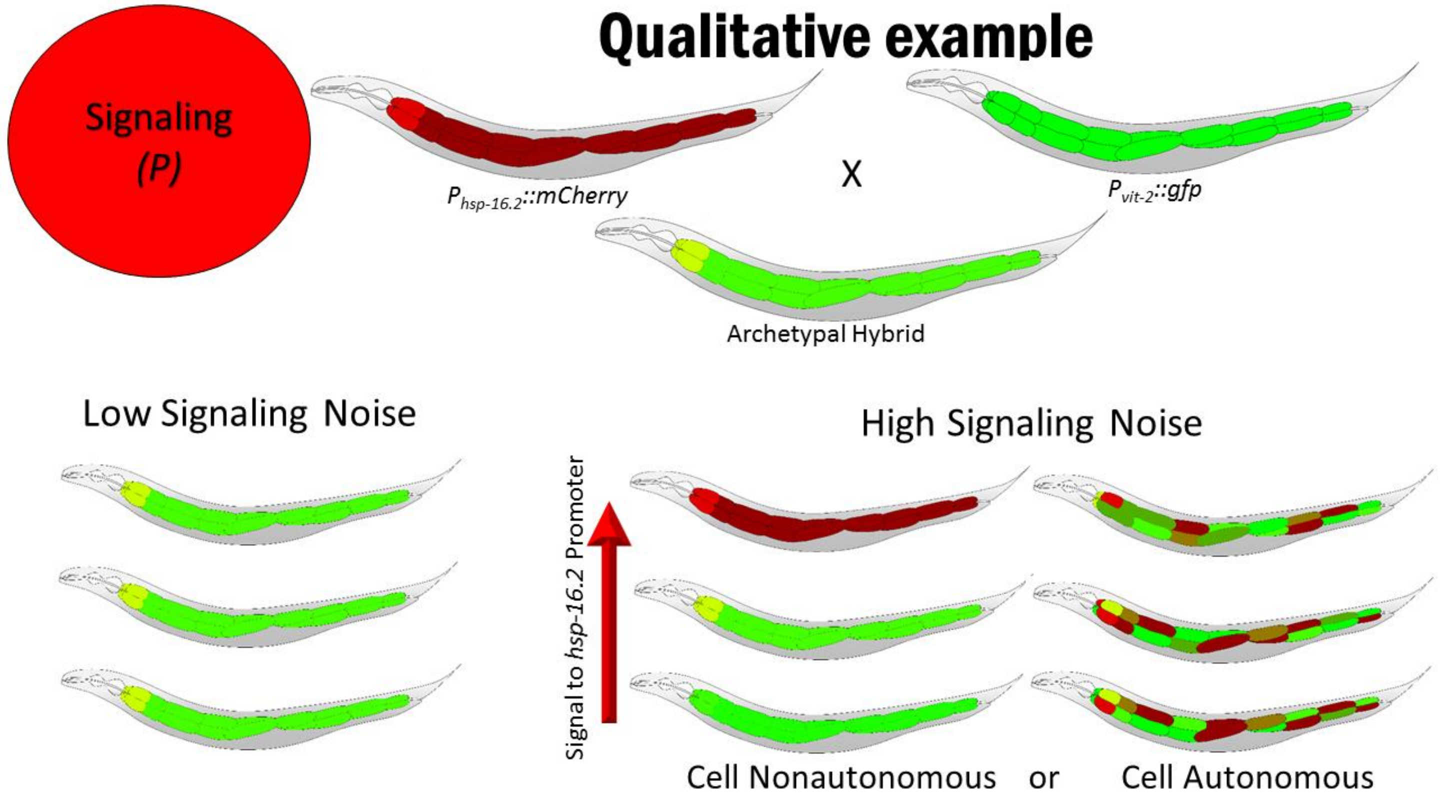

Supplementary Figure 3. Generation of animals to quantify protein expression capacity. Cartoon shows how we generated animals for Type II experiments to examine gene expression capacity. Top section shows two homozygous animals crossed to create an archetypal hybrid. As in Supplementary Fig. 2, each archetypical hybrid of two distinct reporters has its own stereotyped pattern and particular slopes (ratiometric setpoints) for each pair of distinct reporter genes. Bottom Panels show what animals with small differences in *G* would look like (left), what animals with cell nonautonomous differences would look like (center, a cartoon approximation; see Fig. 4 for actual images), and (right) what cell autonomous differences in *G* would look like. All of these images are assuming intrinsic noise and signaling noise are constrained. Again, we use this assumption in these figures, because large amounts of intrinsic noise would occlude our ability to detect signaling noise in a two-signal measurement scheme employed in these studies; large amounts of signaling noise would prevent *G* from manifesting as a dominant axis of variation. The cartoons are intended to show the correct ratiometric setpoints, but different absolute values for the whole intestine for cell nonautonomous and for individual cells that randomly vary in *G*.

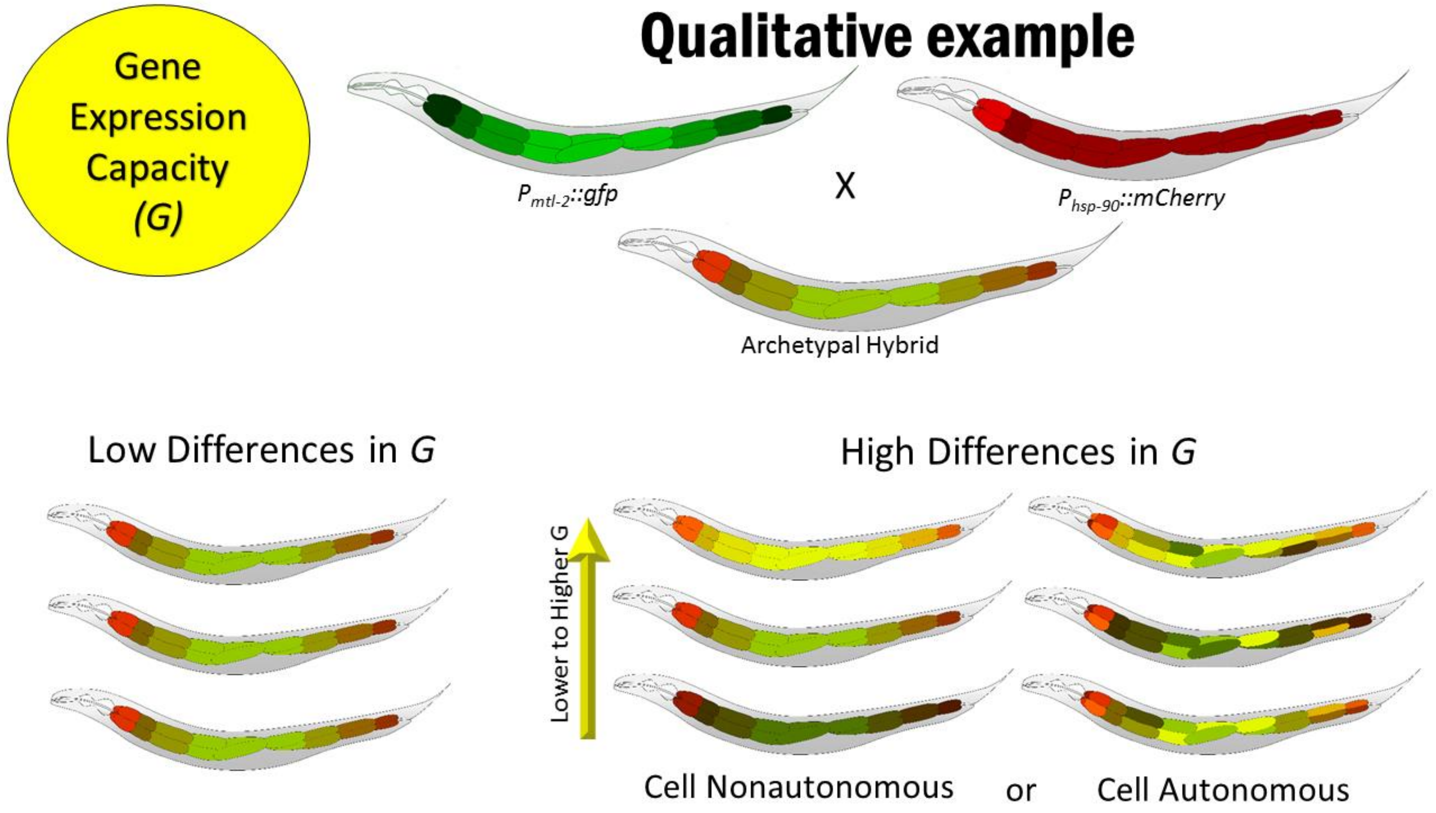

Supplementary Figure 4. Type I experiments supplementary scatterplots.

Scatterplots of two identical promoters with different fluorescent protein outputs expressed from homologous chromosomes (type I experiment). Far left scatterplots show all cells measured in a given experiment. Scatterplots on the right show expression of reporters in the cells from particular intestine rings. Each reporter signal is listed relative to the particular axes of x and y each is labeling. Scale is arbitrary fluorescence signal units. One of at least three repetitions per reporter gene pair is shown.

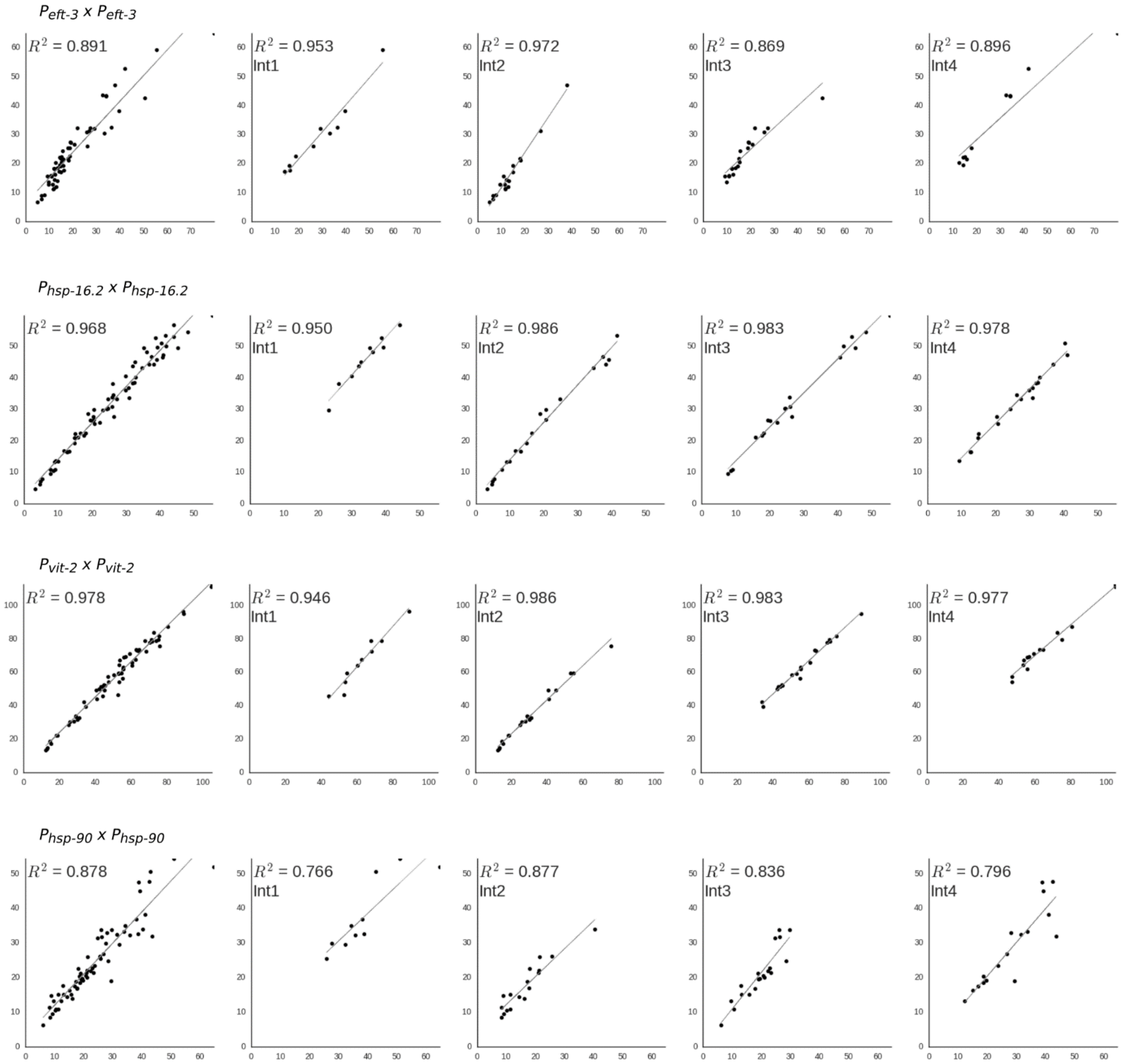

<u>Supplementary Figure 5. Type I experiments supplementary boxplots.</u> Boxplots of correlated and uncorrelated variation for $P_{hsp\text{-}16.2}$, $P_{eft\text{-}3}$, $P_{vit\text{-}2}$ and $P_{\text{-}hsp\text{-}90}$ based reporter genes in intestine cells in rings 1-4. y axis is unitless η^2. In Type I experiments uncorrelated variation arises from stochastic noise of transcription/translation or variable allele access – $\eta^2(\gamma)$ (colored blue as in Figure 1); correlated variation is a combined result of variation in gene expression capacity $\eta^2(G)$ and variation in pathway activation $\eta^2(P)$ (colored orange because the terms are combined). The boxplots consist of data for each cell type from three independent experiments quantifying cells from ten animals in each experiment. Intrinsic noise was significantly different than extrinsic noise ($\eta^2(G)$ + $\eta^2(P)$) in all cases, indicated via $p < 0.05$ via t-test.

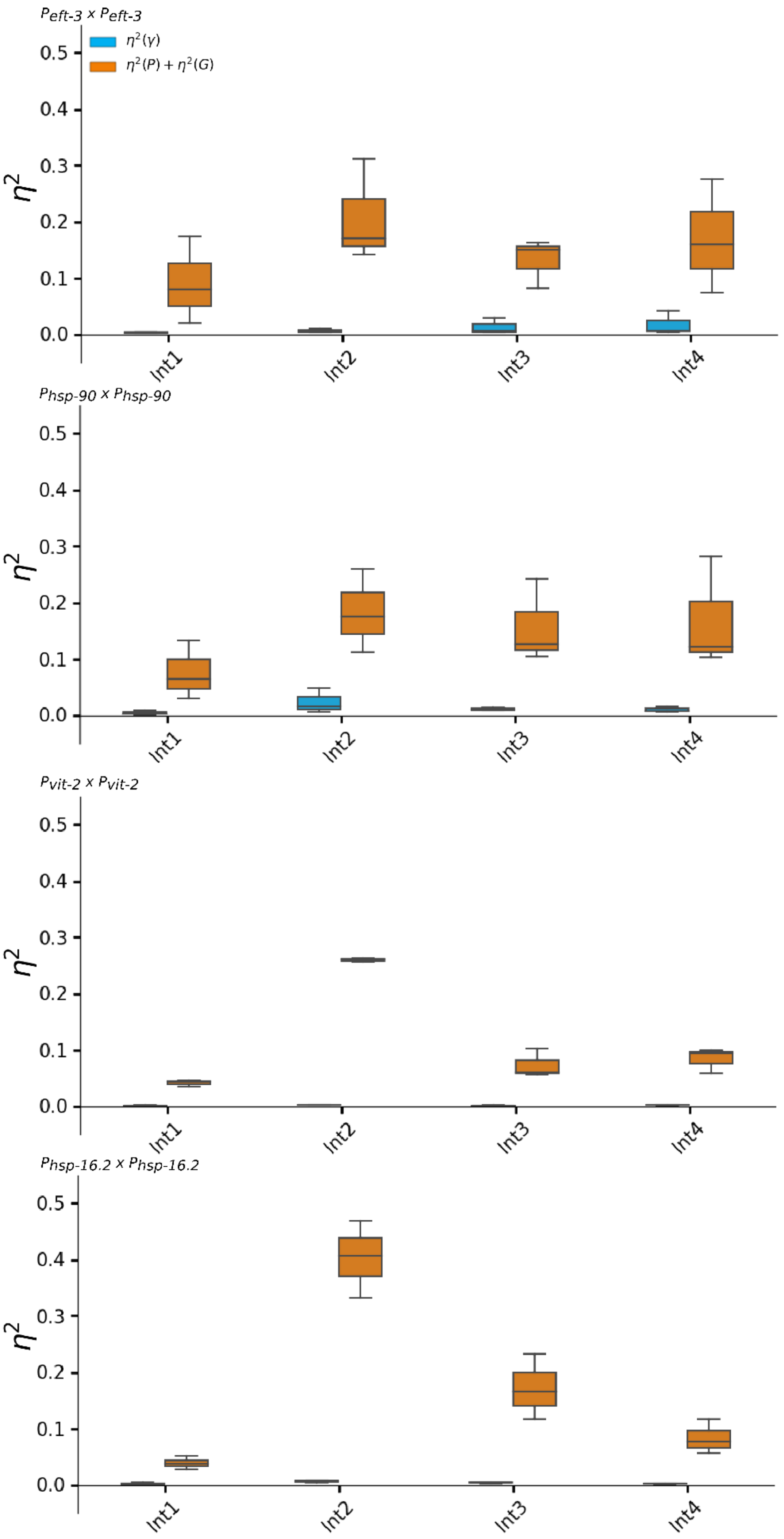

Supplementary Figure 6. Evidence for splitting cells by fate for analysis in Type II experiments.

Scatterplots of expression of two reporters from the different reporter genes, P_{hsp-17} (x axis) and P_{mtl-2} (y axis), grouped by ring (one through four) or combined in one plot (right panel). Cell fate determines ratiometric setpoint for expression of two distinct genes. Specifically, because each gene has a gene-specific stereotypical anterior-posterior expression pattern in the intestine, every gene pair measured has its own particular slope in each cell type (ring), which we refer to as a ratiometric setpoint (see also Supplementary Fig. 2). When cells are not split by fate, correlation of expression is quite low. Correlation of expression is much higher when cells are grouped by fate. Data shown is from an independent experiment quantifying the aforementioned reporters in intestine cells located in intestine rings one through four in ten animals.

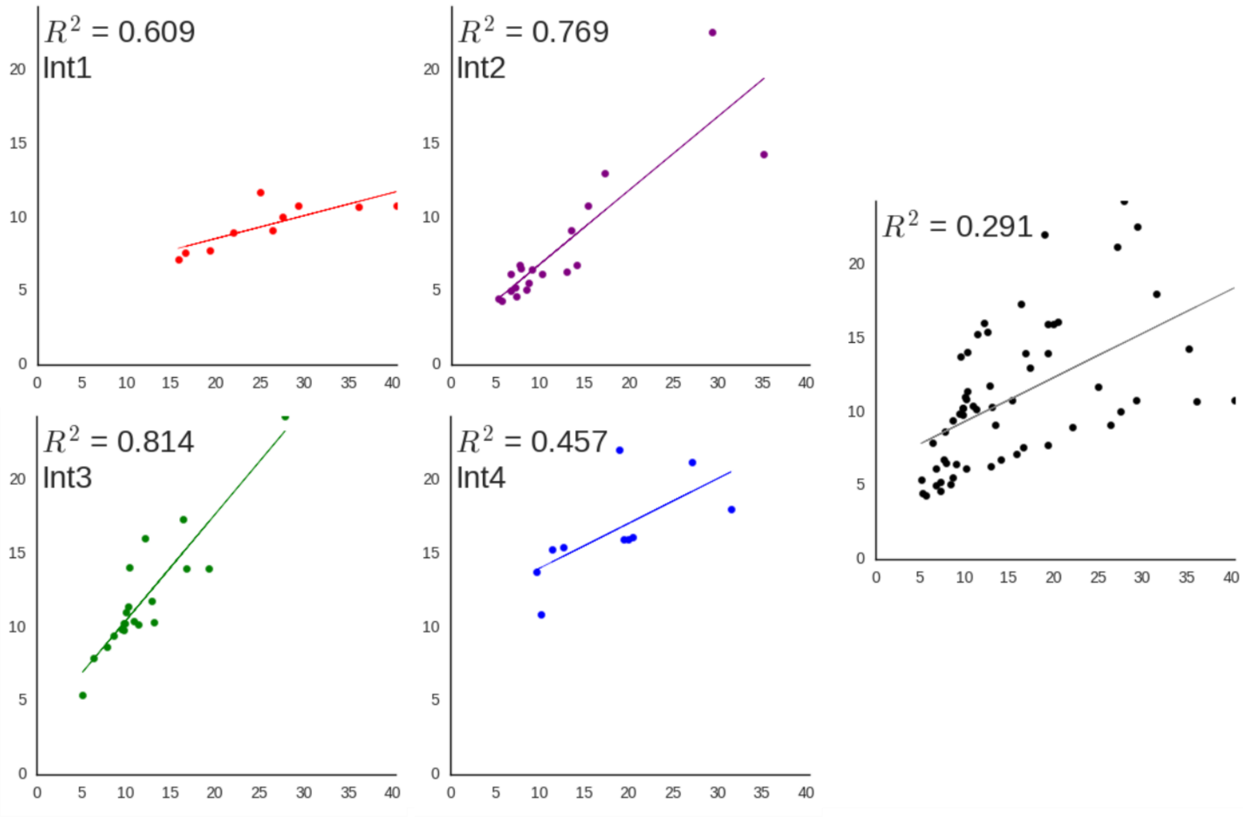

Supplementary Figure 7. Evidence for correct regulation of *eft-3* and *vit-2* expression after heat shock. In response to heat shock, animals decrease progeny production and increase expression of chaperones and *eft-3.* Here, we heat shocked animals to determine if the reporter genes would respond to the heat shock as expected. The scatterplot shows $P_{eft\text{-}3}$::GFP and $P_{vit\text{-}2}$::mCherry expression at whole animal level with (red dots) and without heat shock (blue dots). For heat shock (red dots), day 1 adults were incubated for 1hr at 35ºC and imaged 24 hours later together with age-matched non heat shocked animals (blue dots). While the correlated variation is still the dominant source of variation among individuals in each population, the shifts in expression caused by heat shock are clearly visible. The directionality of the population expression shifts caused by the heat shock are indicated by black arrows annotated with the name of the gene for which they are indicating an expression level shift. **a)** A scatter plot of signaling changes in *vit-2* and *eft-3* reporter genes in response to heat shock from an individual experiment is shown. **b)** Boxplots quantifying average expression levels from individual experiments are shown. Non-heat shocked *vit-2* reporter signal is blue and heat shocked *vit-2* reporter signal is orange; non-heat shocked *eft-3* reporter signal is green and heat shocked *eft-3* reporter signal is red. The boundary of the box closest to zero indicates the 25th percentile, a line within the box marks the median, and the boundary of the box farthest from zero indicates the 75th percentile. Whiskers above and below the box indicate the 90th and 10th percentiles. Diamonds indicate outliers. Asterisks indicate $p < 0.05$ via Mann-Whitney U; except for *vit-2* on the far right, for which $p = 0.057$. Left panel measured 100 non-heat shocked & 105 heat shocked animals, middle panel measured 34 non-heat shocked & 32 heat shocked animals, and right panel measured 99 non-heat shocked & 84 heat shocked animals.

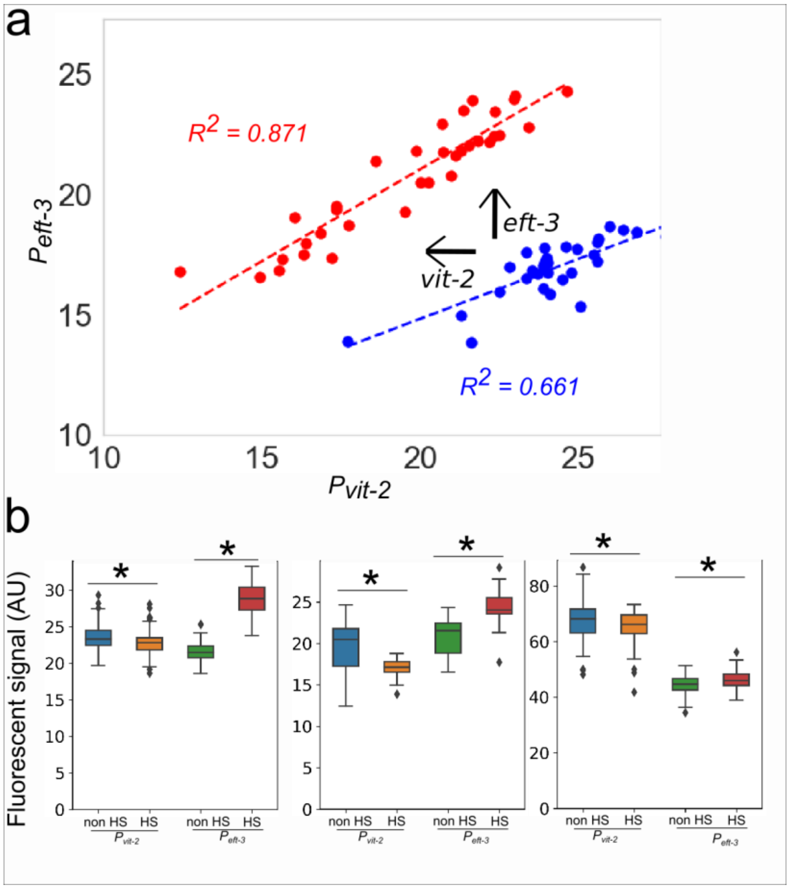

Supplementary Figure 8. Type II experiments supplementary scatterplots.

Scatterplots of expression of two distinct reporter genes (type II experiment). Far left scatterplots show all cells measured in a given experiment. Scatterplots on the right show expression of reporters in the cells from particular intestine rings. Each reporter signal is listed relative to the particular axes of x and y each is labeling. One of at least three repetitions per reporter gene pair is shown.

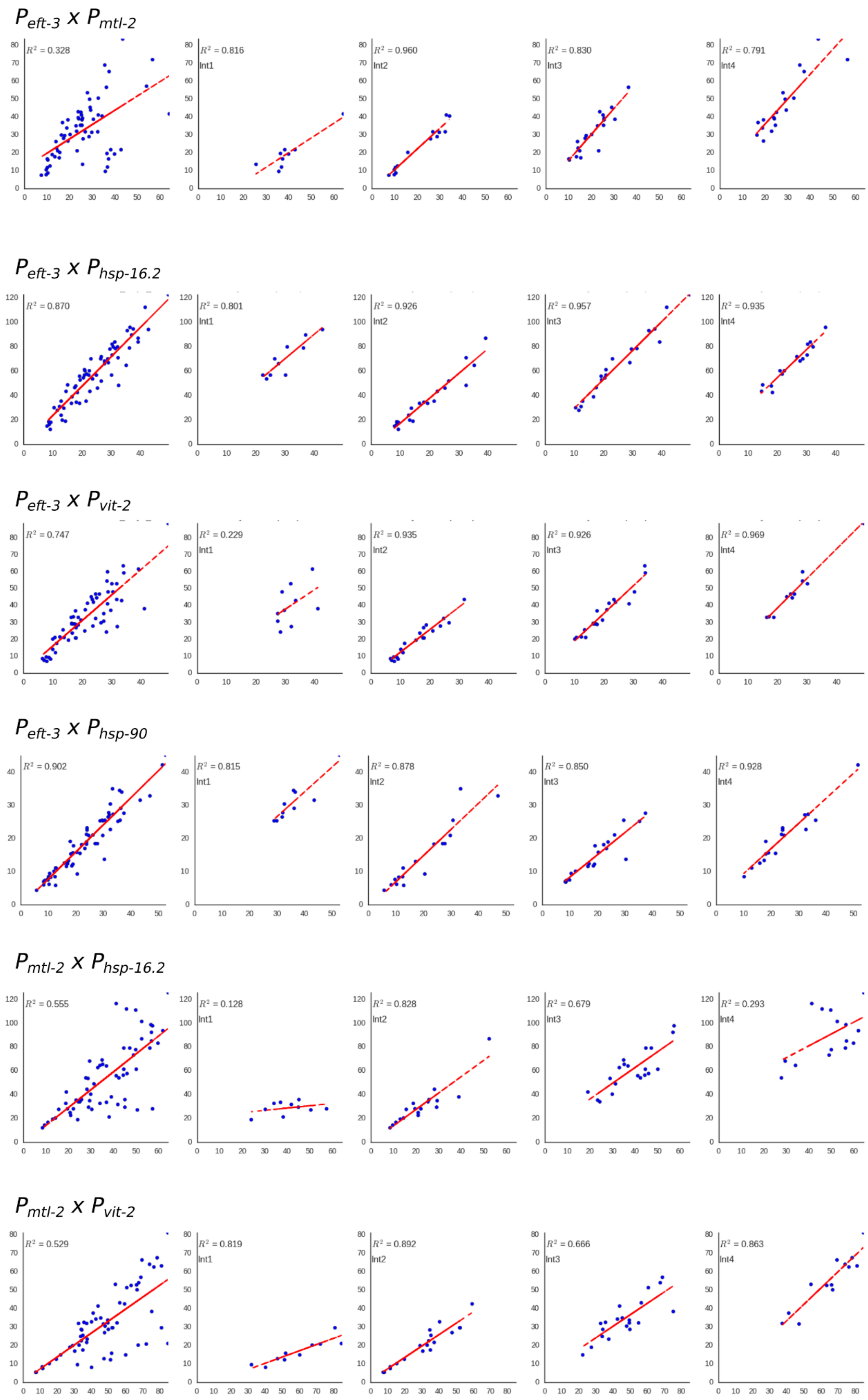

Type II experiment scatterplots Part II

$P_{mtl\text{-}2}$ x $P_{hsp\text{-}90}$

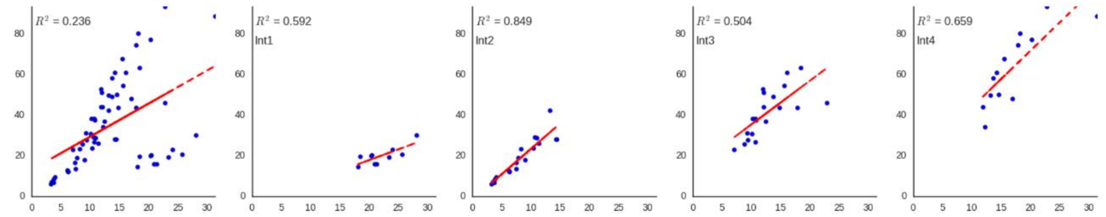

$P_{hsp\text{-}16.2}$ x $P_{vit\text{-}2}$

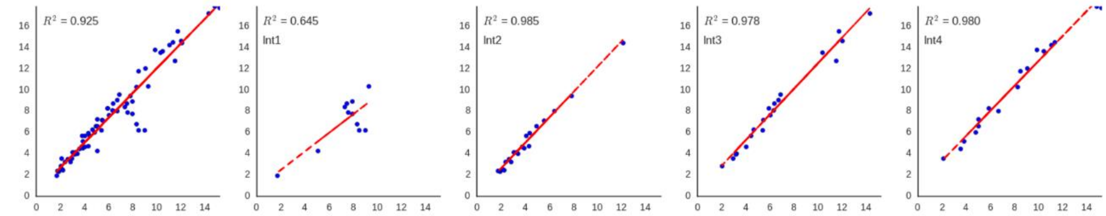

$P_{hsp\text{-}16.2}$ x $P_{hsp\text{-}90}$

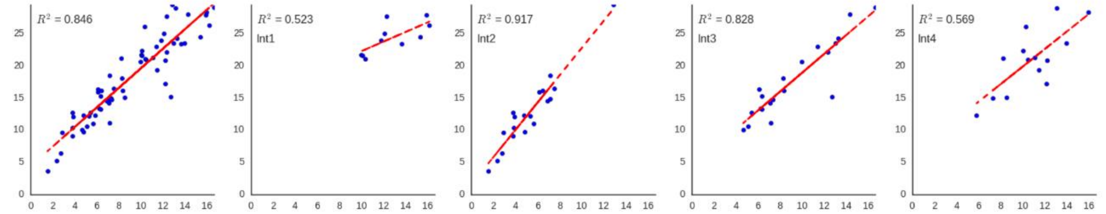

$P_{hsp\text{-}90}$ x $P_{vit\text{-}2}$

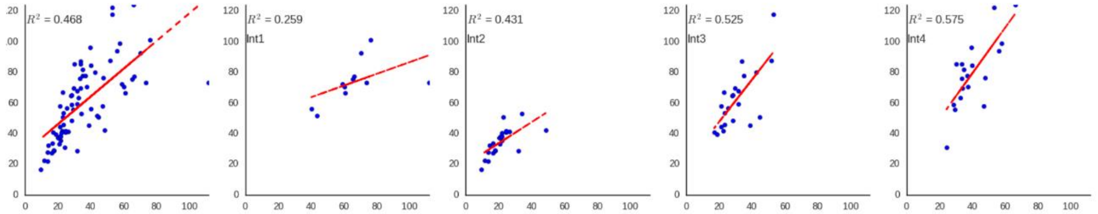

$P_{hsp\text{-}17}$ x $P_{mtl\text{-}2}$

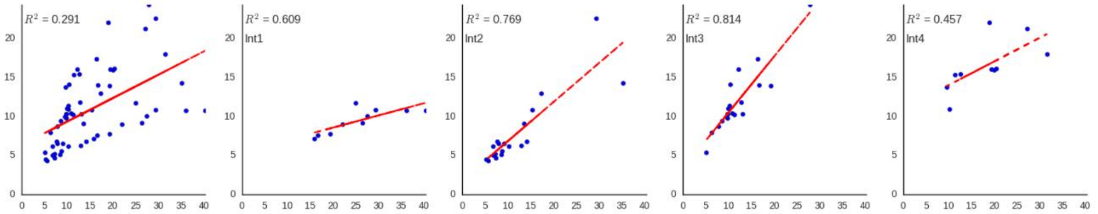

$P_{hsp\text{-}17}$ x $P_{hsp\text{-}16.2}$

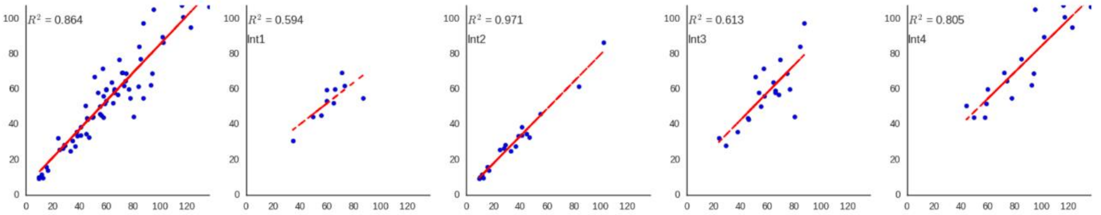

Type II experiment scatterplots Part III

$P_{hsp\text{-}17}$ x $P_{vit\text{-}2}$

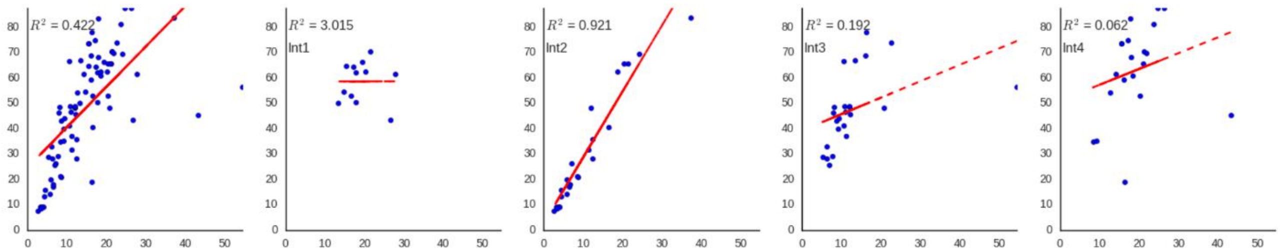

$P_{hsp\text{-}17}$ x $P_{hsp\text{-}90}$

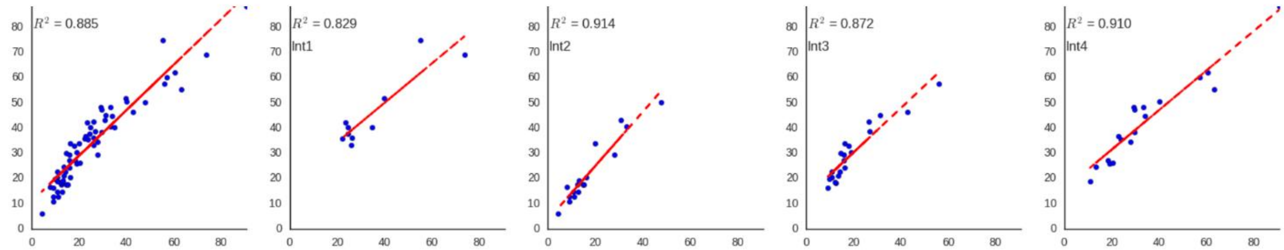

$P_{hsp\text{-}17}$ x $P_{eft\text{-}3}$

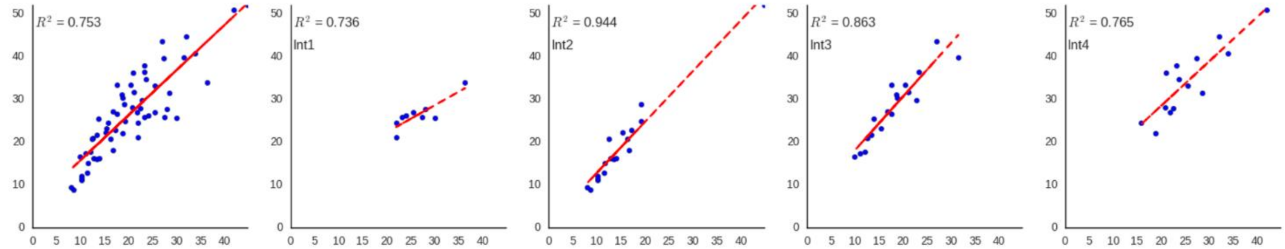

EMR-1 x $P_{hsp\text{-}16.2}$

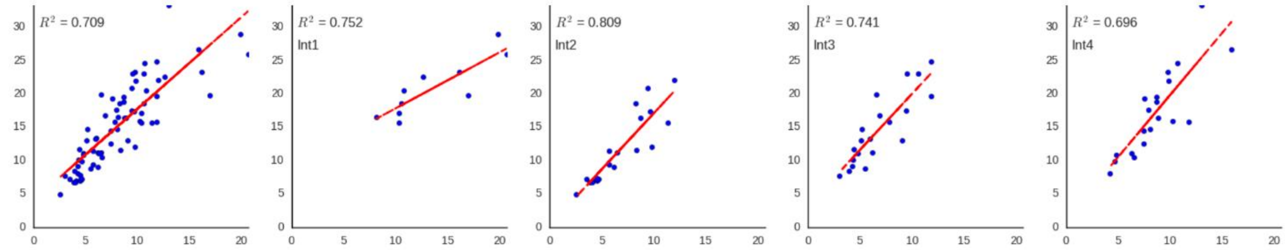

Supplementary Figure 9. Type II experiments supplementary boxplots.

Stochastic noise $\eta^2(\gamma)$ (red boxes), variation in pathway activation $\eta^2(P)$ (blue boxes) and variation in gene expression capacity $\eta^2(G)$ (yellow boxes) for $P_{hsp\text{-}16.2}$, $P_{vit\text{-}2}$, $P_{eft\text{-}3}$ and $P_{hsp\text{-}90}$ for cells in intestine rings 1-4. y axis is unitless η^2. The boundary of the box closest to zero indicates the 25th percentile, a line within the box marks the median, and the boundary of the box farthest from zero indicates the 75th percentile. Whiskers above and below the box indicate the 90th and 10th percentiles. Diamonds indicate outliers. These boxplots are composed of data from three independent experiments quantifying expression from cells from ten animals per experiment. Asterisks indicate statistical significance of $p < 0.05$ analyzed by one-way ANOVA with post-hoc Tukey's HSD test.

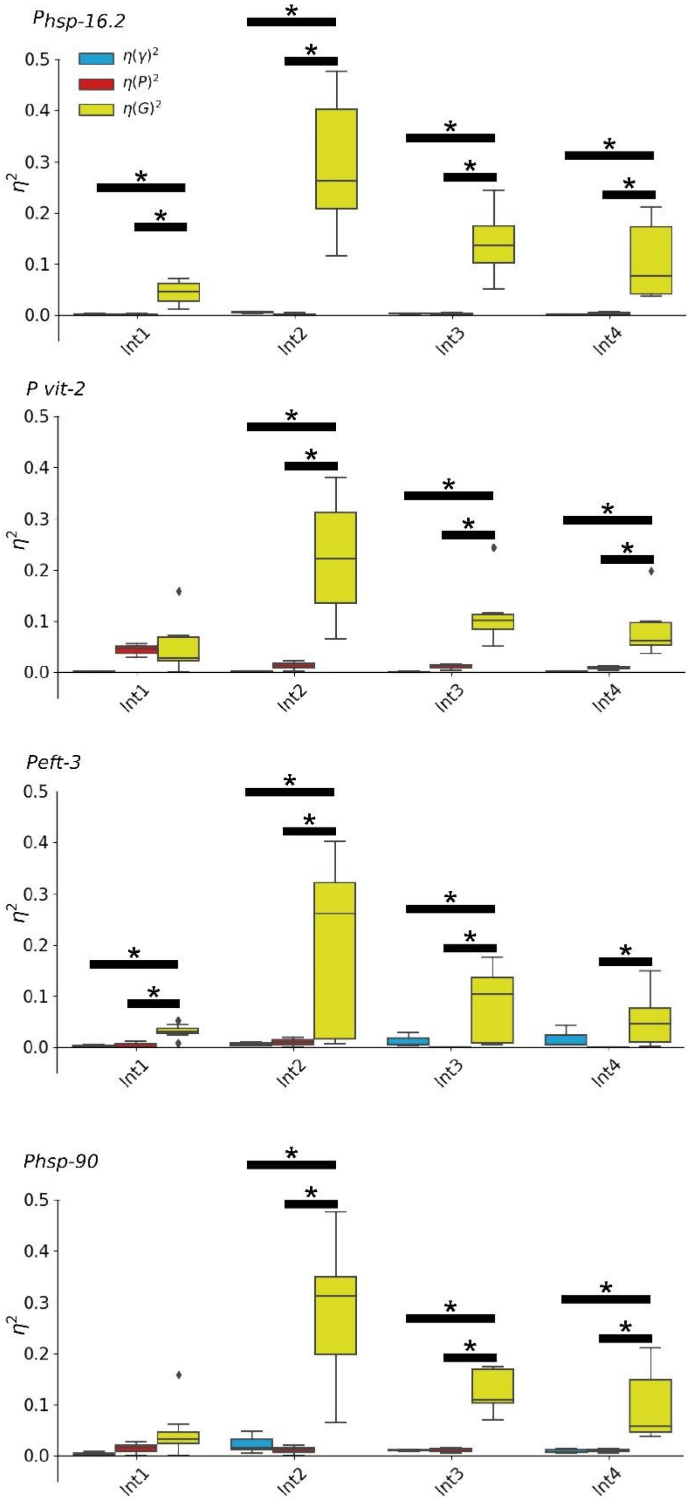

Supplementary Figure 10. Type II experiments supplementary boxplots (no promoter splitting)

Boxplots of correlated and uncorrelated variation for different gene pairs in cells in intestine rings 1-4. y axis is unitless η^2. Uncorrelated variation combines stochastic noise of transcription/translation or variable allele access – $\eta^2(\gamma)$, and variation in pathway activation $\eta^2(P)$ (purple boxes as that is the merge of the colors of each component, listed in Figure 1). Correlated variation results from variation in gene expression capacity $\eta^2(G)$ (yellow boxes as $\eta^2(G)$ is yellow in Figure 1). The boundary of the box closest to zero indicates the 25th percentile, a line within the box marks the median, and the boundary of the box farthest from zero indicates the 75th percentile. Whiskers above and below the box indicate the 90th and 10th percentiles. Diamonds indicate outliers. These box plots are composed of data from three independent experiments quantifying expression from two cells per ring per animal for at least ten animals per experiment. Asterisks indicate $p < 0.05$ via Mann-Whitney U test. Values are calculated from cells from thirty animals per gene pair.

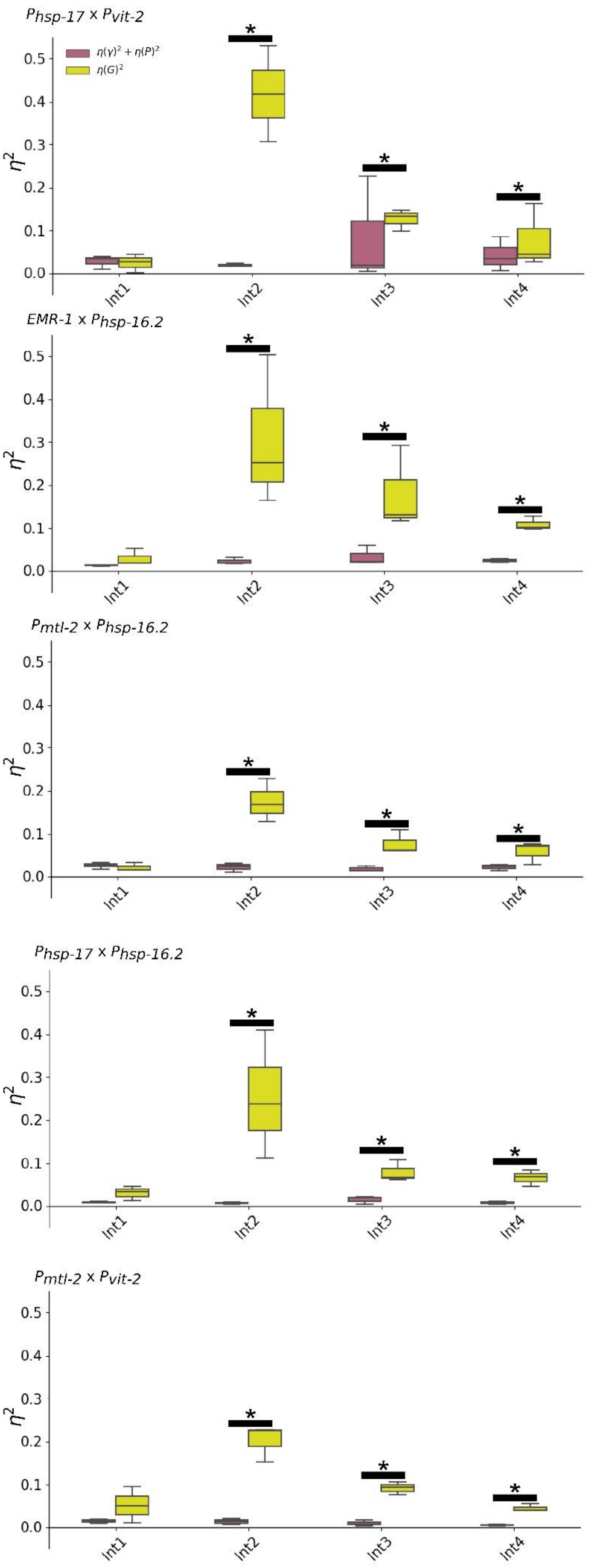

Supplementary Figure 11. Additional experiments quantifying expression of nuclear proteins. Replicate experiments of Fig. 4b, showing 3-dimensional scatterplots of nuclear fusion proteins LMN-1::BFP (single intergenic copy, Chromosome I), HIS-72::GFP (knockin) and EMR-1::mCherry (single intergenic copy, chromosome II). The data in these scatter plots were generated by measuring individual intestine cell expression levels of all three reporter genes from ten animals in two additional independent experiments. **a&c** show 3d scatter plots of the two additional replicate experiments. **b&d** show corresponding series of three 2d scatter plots for each pairwise comparison from the data from each of these two replicate experiments. A positive correlation is apparent from these scatter plots.

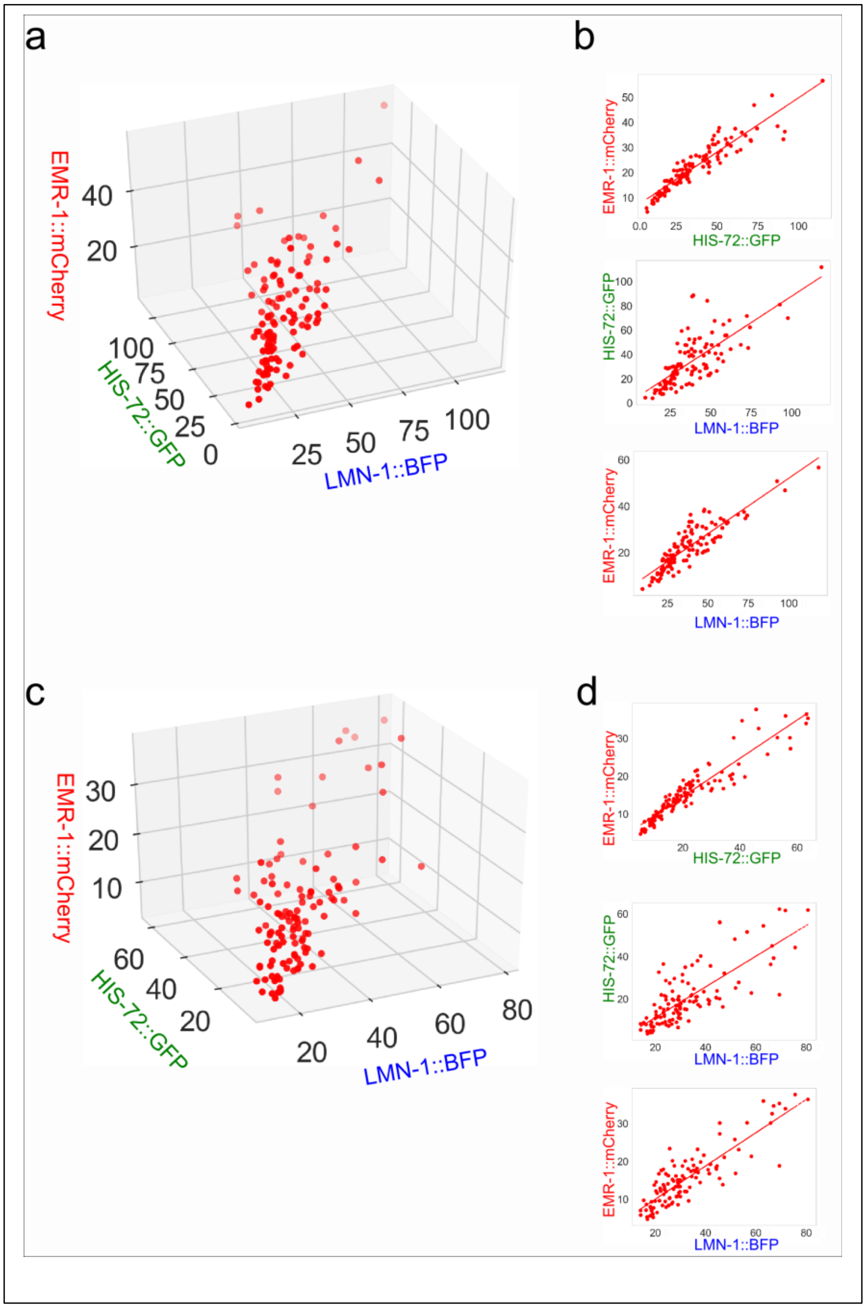

<u>Supplementary Figure 12. Animals that respond better to heat shock produce and maintain timer protein better.</u> a. Boxplots of protein age in nine L1 and sixteen 2-day old adult animals. The boundary of the box closest to zero indicates the 25th percentile, a line within the box marks the median, a dash within the box marks the average, and the boundary of the box farthest from zero indicates the 75th percentile. Whiskers above and below the box indicate the 90th and 10th percentiles. Dots indicate outliers. **b.** Scatterplot of whole animal average values fluorescent timer protein expressed from *eft-3* promoter ($P_{eft\text{-}3}$*::timer*). Blue dots represent non-heat shocked animals and red dots represent heat shocked animals. Linear trend for young and old protein fractions indicates that bright animals are better at both protein production and maintenance. **c**. Boxplots of whole-animal abundance of young and old fraction of timer protein with and without heat shock in arbitrary units (tiff counts on y axes; diamonds are outliers). Young protein in non-heat shocked animals is shown in blue, and for heat shocked animals it is orange; old protein is shown in non-heat shocked animals as green and for heat shocked animals it is red. The three plots correspond to three independent biological replicates. Left panel measured 70 non-heat shocked & 104 heat shocked animals, middle panel measured 34 non-heat shocked & 35 heat shocked animals, and right panel measured 91 non-heat shocked & 74 heat shocked animals. Asterisks indicate $p < 0.05$ via Mann-Whitney U test.

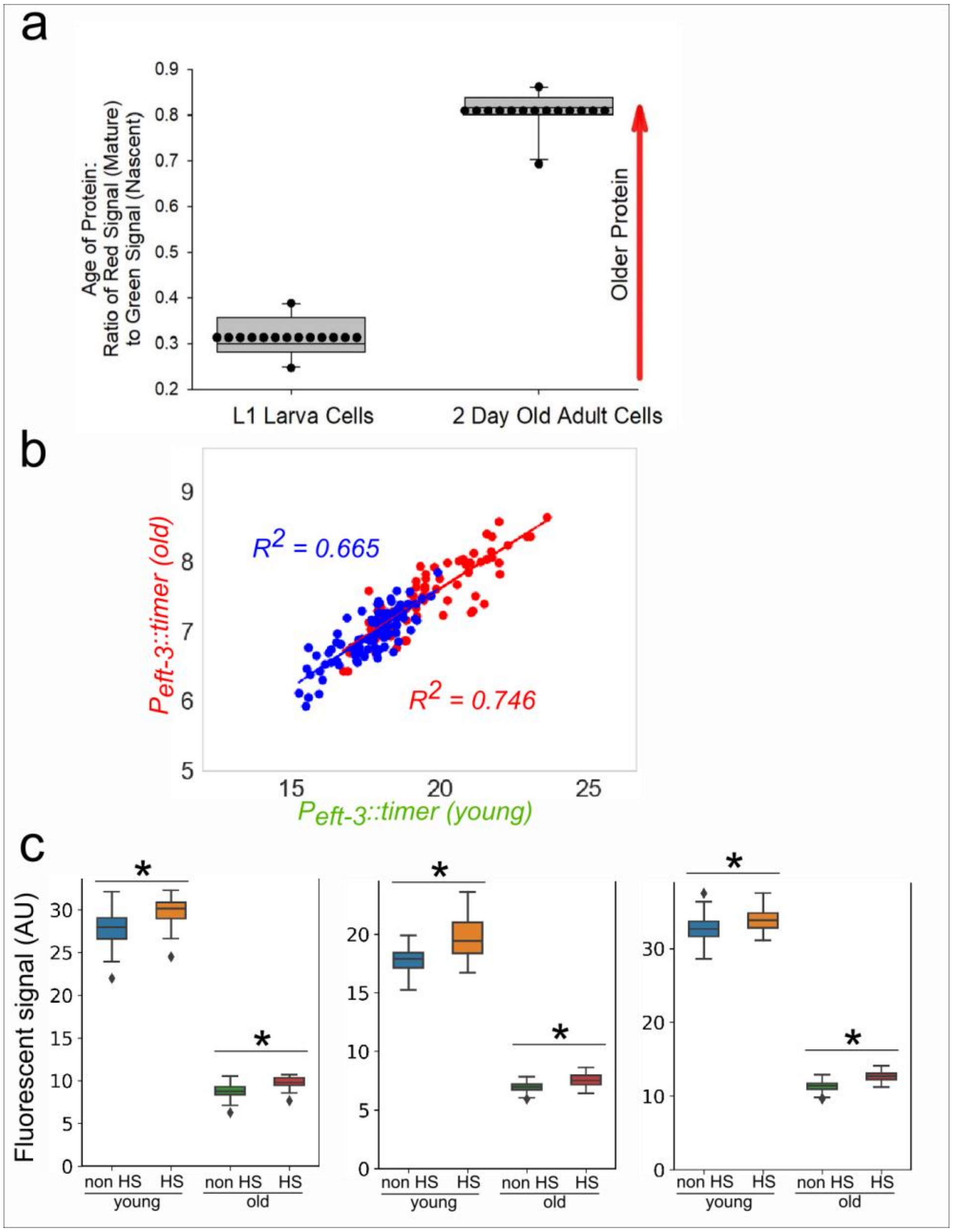

Supplementary Note 1: Defining Pathway Output and Expression Capacity

For quantitative analysis we adapted the analytical framework developed previously by Coleman-Lerner et al and briefly described below. We considered the production of fluorescent proteins (e.g. GFP and mCherry) in individual cells to be a measure of activities of the promoters that regulate their expression. Furthermore, to understand cell-to-cell and animal-to-animal differences in activities of various promoters we considered the amount of fluorescent protein produced to be the product of two subsystems: pathway and expression.

"Pathways" is the first subsystem. For instance, for a heat shock inducible promoter $P_{hsp\text{-}16.2}$, the input to pathway is the activation of transcription factors, e.g. HSF-1, that bind to *hsp-16.2* promoter, and the output is the activation of the inducible promoter that drives the fluorescent protein. Pathway output depends on the summed activity of upstream, DNA-bound transcription factors.

For a given cell *i,* pathway output is proportional to P_i, the time-averaged of the level of pathway activation. We separate P_i as $P_i = L_i + \lambda_i$ where L_i is the expectation value of P_i for a given cell type in a cohort of isogenic age-matched animals, and λ_i is the stochastic fluctuation term for that cell. We refer to L_i as the pathway power. The pathway "power" is a function of the activities of upstream signaling molecules and transcription factors that lead to activation of the promoter. We refer to cell-to-cell differences in P_i as variation in pathway power, and we describe differences that result from the stochastic fluctuation term λ_i as transmission noise. Thus, we can treat the output of the cell as being decomposed into an expectation value, which depends on the number and activity of the molecules that comprise the pathway, and a stochastic fluctuation that occurs because of the inherent randomness of the activity of the pathway, as well as its composition over the time of the experiment. Different cells may have different pathway capacities, and therefore, can have different values of L_i.

The second subsystem consists of the sequence of events from gene transcription through protein translation. We call this subsystem expression. It includes transcriptional initiation, elongation, mRNA maturation, nuclear export, and mRNA translation and degradation (of protein and mRNA). We use our data to measure cell-to-cell variation in expression, but we cannot isolate the contribution of each part of the expression machinery to the overall variation.

The output of the expression subsystem is the total amount of the fluorescent reporter protein, and the input is proportional to the level of promoter activity (the output of the pathway subsystem). For analytical

purposes, we assume here that the expression per unit of input is independent of the level of input. For cell *i,* we describe the expression per unit input as the variable E_i, where E_i is given by the sum of G_i and γ_i. The quantity G_i is the expectation value of the expression per unit input for cell *i,* and this quantity may vary from cell to cell. We refer to G_i as the “expression capacity” for cell *i*, and we refer to the cell-to-cell differences in G_i as “variation in expression capacity”. The quantity γ_i is the stochastic fluctuation that occurred in cell *i*, and we refer to differences that result from this stochastic fluctuation term as expression noise.

Using this model, we described the total amount of GFP, y_i, in cell *i* as

$$y_i = (L_i + \lambda_i) \text{ x } (G_i + \gamma_i) \text{ x } \Delta\mathrm{T} \qquad (1)$$

where ΔT is the time of reporter protein production. For heat inducible promoter we can consider ΔT as the time since the heat shock. For constitutive promoters like *vit-2* we cannot precisely measure what fraction of the observed reporter protein pool was produced in the same time frame. Thus, we started this work under steady state assumption that reporter protein synthesis and degradation are balanced in young adult animals. In that case reporter proteins level directly mirror activity of the promoters and expression capacity. Highly correlated expression of the examined promoters suggests that this assumption is applicable and acceptable.

Supplementary Note 2: Extracting Pathway and Expression Capacity Information from the Data

Variance and Covariance

We define the variance of a given quantity *x* for a population of cells as

$$\sigma^2 = \frac{1}{N}\sum(x_i - \bar{x})^2 \qquad (2)$$

$$= \overline{(x_\iota - \bar{x})} \qquad (3)$$

where *N* is the number of cells in the population. Since, we examined homologous cells of *C. elegans* nematodes, this number is equal to the number of animals in the population. We use the overbar symbol ($\bar{x}$) to represent the population average. Similarly we define the covariance, *Cov*(*x*,*y*), of two quantities *x* and *y* as

$$Cov(x, y) = \frac{1}{N}\sum(x_i - \bar{x})(y_i - \bar{y}) \qquad (4)$$

$$= \overline{(x_\iota - \bar{x})(y_\iota - \bar{y})} \qquad (5)$$

The covariance will be non-zero if x and y are correlated (or anti-correlated).

The correlation coefficient, $\rho(x,y)$, is the covariance scaled by the standard deviations. Specifically

$$\rho(x,y) = \frac{Cov(x,y)}{\sigma(x)\sigma(y)} \tag{6}$$

Supplementary Note 3: Calculating Variance and Covariance of *G*, *γ*, *L*, and *λ*

As discussed above we assumed that in standard conditions, the average pathway output (P) for a given cell has a pathway power L associated with it, along with a stochastic term, λ. Thus, we have $P=L+\lambda$. Similarly, the expression subsystem of the cell (E) contains a capacity term G and a stochastic term γ.

Thus, the amount of fluorescent reporter protein (y_i) produced for a given cell i is the product of the terms

$$y_i = (L_i + \lambda_i) \times (G_i + \gamma_i) \text{ x } \Delta\mathrm{T} \tag{7}$$

$$= (L_i G_i + L_i \gamma_i + \lambda_i G_i + \lambda_i \gamma_i) \times \Delta\mathrm{T} \tag{8}$$

Since the expectations of the stochastic fluctuation terms are zero, and since their fluctuations are uncorrelated to other terms, we expect that the population average of y_i reduces to the population average of the quantity $L_i G_i \Delta T$.

To calculate the average of y, we re-write the product L_i x G_i in terms of population averages and deviations from the average. For these two terms, we use a capital delta (Δ) to represent a deviation from a population average. We get

$$L_i = \bar{L} + \Delta L_i \tag{9}$$

$$G_i = \bar{G} + \Delta G_i \tag{10}$$

We then calculate the population average of y to be

$$\bar{y} = \frac{\Delta}{T}\sum(L_i \times G_i) \quad (11)$$

$$= (\bar{L} \times \bar{G} + Cov(L,G))\Delta T \quad (12)$$

where we used equation (4) for the definition of covariance and the fact that *ΔG=ΔL=0*. Thus the average number of GFP molecules is the product of *L* and *G* plus an extra term to account for their correlation.

To calculate the variance on *y*, we re-write equation (7) in terms of equations (9) and (10). We get

$$y_i = (\bar{L} + \Delta L_i + \lambda_i)\Delta T \times (\bar{G} + \Delta G_i + \gamma_i) = \quad (13)$$

$$= \left(1 \frac{\Delta L_i}{\bar{L}} + \frac{\lambda_i}{\bar{L}}\right) \times \left(1 + \frac{\Delta G_i}{\bar{G}} + \frac{\gamma_i}{\bar{G}}\right) \times \bar{G} \times \bar{L} \times \Delta T \quad (14)$$

$$= \left(1 + \frac{\Delta L_i}{\bar{L}} + \frac{\lambda_i}{\bar{L}}\right) \times \left(1 + \frac{\Delta G_i}{\bar{G}} + \frac{\gamma_i}{\bar{G}}\right) \times \bar{y}(1 - \frac{Cov(L,G)\Delta T}{\bar{y}}) \quad (15)$$

$$\approx y \times (1 + \frac{\Delta L_i}{\bar{L}} + \frac{\lambda_i}{\bar{L}} + \frac{\Delta G_i}{\bar{G}} + \frac{\gamma_i}{\bar{G}}) \quad (16)$$

where we used equation (12) to make the substitution for $\bar{L} \times \bar{G} \times \Delta T$ in going from equation (14) to (15); and where we have dropped, in going from equation (15) to (16) all higher order terms of fractional deviations from the means. These higher order terms are various products of $\frac{\Delta L_i}{\bar{L}}$, $\frac{\lambda_i}{\bar{L}}$, $\frac{\Delta G_i}{\bar{G}}$, and $\frac{\gamma_i}{\bar{G}}$. We assume that each of the higher order terms is small relative to the lower order terms that are retained in equation (16), and therefore we have neglected them. The term $\frac{Cov(L,G)\Delta T}{\bar{y}}$ is second order, and has also been neglected. Discussion of the magnitude of the error introduced by this approximation is provided in Coleman-Lerner et al.

We use equation (16) to calculate the variance on the number of GFP molecules using the definitions of variance and covariance above. We get

$$\frac{\sigma^2(y)}{\bar{y}^2} = \frac{\sigma^2(L)}{\bar{L}^2} + \frac{\sigma^2(\lambda)}{\bar{L}^2} + \frac{\sigma^2(G)}{\bar{G}^2} + \frac{\sigma^2(\gamma)}{\bar{G}^2} + 2\rho(L,G)\frac{\sigma(L)}{\bar{L}}\frac{\sigma(G)}{\bar{G}} \quad (17)$$

where *ρ(L,G)* is the correlation coefficient between *L* and *G*.

The correlation coefficient is always between -1 and 1, and a value of 0 corresponds to no correlation. There may be no correlation between L and G, but, for example, one possibility that would lead to a correlation is that cells with higher expression capacity may have stronger or weaker pathway output, perhaps because they have higher amounts of positive regulators or negative regulators of the pathway, respectively. This would show up as a positive or negative value for $\rho(L,G)$, respectively.

We do not expect that the stochastic fluctuations λ or γ are correlated to any other variables, and thus the terms $\rho(L,\lambda)$, $\rho(L,\gamma)$, $\rho(\gamma,G)$, $\rho(\lambda,G)$, *and* $\rho(\lambda,\gamma)$ were omitted from equation (17).

All the variances in equation (17) are expressed as fractions of the means squared. We define the variables

$$\eta(L) \equiv \sigma(L)/\bar{L} \qquad (18)$$

$$\eta(\lambda) \equiv \sigma(\lambda)/\bar{L} \qquad (19)$$

$$\eta(G) \equiv \sigma(G)/\bar{G} \qquad (20)$$

$$\eta(\gamma) \equiv \sigma(\gamma)/\bar{G} \qquad (21)$$

The quantity η is simply the width of different distributions expressed as a fraction of their means. For the stochastic variables λ and γ, we will refer to $\eta(\lambda)$ and $\eta(\gamma)$ as the noise associated with those quantities, and for the non-stochastic variables L and G, we will refer to $\eta(L)$ and $\eta(G)$ as the "variation."

We quantified cell-to-cell variation in system output using normalized variance ($\eta^2 = \sigma^2/\mu^2$) rather than noise strength (σ^2/μ), a measure others have used to describe deviations from purely stochastic Poisson-type biological processes. We used normalized variance for a number of reasons. First, we found that most of the cell-to-cell differences in system behavior we reported are not due to stochastic differences in signal transmission or gene expression, as described in the main text. Second, use of η^2 allowed examination of different amounts of variation in terms of the fraction of the mean. Third, and most important, because η^2 is unitless, it allowed direct comparison of different measurements, for example, from different fluorescent proteins, and it allowed the definition of total variation as the sum of individual sources of variation plus additional terms to account for correlations.

We re-write equation (17) in terms of η as

$$\eta^2(y) = \eta^2(P) + \eta^2(G) + \eta^2(\gamma) + 2\rho(L,G)\eta(L)\eta(G) \quad (22)$$

We have written $\eta^2(P)$ for $\eta^2(L)+\eta^2(\lambda)$ since the data will not be able to distinguish the cell-to-cell variation in pathway output from the noise or stochastic fluctuations. The term $\eta^2(P)$ is the variation in average pathway output per unit time, which, because pathway output is given by $P\Delta T$ and ΔT is the same for every cell, is identical to variation in pathway output.

To separate $\eta(G)$ from $\eta(P)$ and $\eta(\gamma)$ in the data from the two-promoter, two-color experiments, discussed further below, we introduce the quantity

$$Z(GFP, mCherry) = \frac{\eta^2(GFP)+\eta^2(mCherry)}{2} - \eta(GFP)\eta(mCherry)\rho(GFP, mCherry)$$

$$= \frac{\eta^2(GFP)+\eta^2(mCherry)}{2} - \frac{Cov(GFP,mCherry)}{\overline{GFP} \times \overline{mCherry}} \quad (23)$$

The quantity Z is the average variance divided by the mean square of the two fluorescence signals GFP and mCherry with the correlated part subtracted out. Thus, Z is a measure of the uncorrelated part of the GFP vs mCherry scatter plot. Depending of the type of the experiment (two identical promoters or two different promoters) Z encompasses distinct sources of variation.

Supplementary Note 4: Two Color Variants Driven by the Same Promoter and Intrinsic Noise of Gene Expression

We calculate the correlation coefficient between GFP and mCherry reporter proteins for the case that both genes are expressed in the same cell and with the same promoter as

$$\rho(GFP, mCherry) \equiv \frac{1}{\sigma(GFP)\sigma(mCherry)}\frac{1}{N}\sum_i \Delta(GFP_i)\Delta(mCherry_i) \quad (24)$$

$$= \frac{1}{\eta(GFP)\eta(mCherry)}\frac{1}{N}\sum_i\left(\frac{\Delta L_i}{\bar{L}} + \frac{\lambda_i}{\bar{L}} + \frac{\Delta G_i}{\bar{G}} + \frac{\gamma_{GFP,i}}{\bar{G}}\right)\left(\frac{\Delta L_i}{\bar{L}} + \frac{\lambda_i}{\bar{L}} + \frac{\Delta G_i}{\bar{G}} + \frac{\gamma_{mCherry,i}}{\bar{G}}\right) \qquad (25)$$

where we have used equation (16) for the deviations from the mean, $\Delta(GFP_i)$, and an analogous equation for $\Delta(mCherry_i)$. Since the GFP gene has the same promoter as mCherry, ΔL_i is the same for both color variants. The quantity λ_i is the same since stochastic fluctuations in pathway output occur upstream of the promoters (with the exception of the binding of the transcription factors to each individual copy of the reporter genes, see the end of this section), and the quantity ΔG_i is the same since the proteins are being expressed in the same cell. The stochastic fluctuations in gene expression, γ_i, however, are different for the two color variants and we write them as $\gamma_{GFP,i}$ and $\gamma_{mCherry,i}$ for GFP and mCherry respectively.

The uncorrelated terms drop out of equation (25) when we perform the population average. We do not expect the stochastic fluctuations to be correlated with any other terms, and equation (25) becomes

$$\rho(GFP, mCherry) = \frac{\eta^2(P)+\eta^2(G)+2\rho(L,G)\eta(L)\eta(G)}{\eta(GFP)\,\eta(mCherry)} \qquad (26)$$

Where $\eta^2(P) = \eta^2(L) + \eta^2(\lambda)$ as discussed above.

The quantity $Z(GFP, mCherry)$ as defined in equation (23), gives the contribution of the uncorrelated part of the expression of the two color variants to the average η^2 of the two colors. Only the gene expression noise is uncorrelated, and we get

$$Z(GFP, mCherry) = \frac{\eta^2(\gamma_{GFP})+\eta^2(\gamma_{mCherry})}{2} \qquad (27)$$

$Z(GFP, mCherry)$ is the average of gene expression noise of the two promoters. Since the same promoters are driving the GFP and mCherry genes, we expect equal levels of mRNA for the two variants on average, and therefore the gene expression noise is the same for the GFP and mCherry signal. We get $\eta^2(\gamma_{GFP}) = \eta^2(\gamma_{mCherry}) = \eta^2(\gamma)$ and therefore, from equation (27), $Z(GFP, mCherry) = \eta^2(\gamma)$. Thus, equation (23) gives direct measurement of gene expression noise γ when applied to an experiment with two identical promoters:

$$\eta^2(\gamma) = \frac{\eta^2(GFP)+\eta^2(mCherry)}{2} - \frac{Cov(GFP,mCherry)}{\overline{GFP} \times \overline{mCherry}} \qquad (28)$$

<u>We used this equation (28) for practical calculations of gene expression noise of individual promoters.</u>

We note that for the experiment described above, GFP and mCherry were not driven by the same promoter, but rather by identical *copies* of the same promoter. Therefore, stochasticity in the binding of transcription factors to the DNA and in the subsequent activation of transcription contributes to the uncorrelated part of the total variation. In our *ad hoc* model, these molecular steps are part of the pathway subsystem, and therefore fluctuations in this step should contribute to the transmission noise, $\eta^2(\lambda)$. However, due to experimental limitations of the type of experiment we have just described, the noise caused by these molecular steps is, instead, included in the measure of gene expression noise $\eta^2(\gamma)$.

Supplementary Note 5: Two Color Variants Driven by Different Promoters and Pathway Variation

For this case, the calculation of *ρ(GFP,mCherry)* is the same as in equation (25) above except that the pathway activity is different for the two color variants since different promoters are driving the GFP and mCherry genes. If we assume that the two terms *L* and *λ* are uncorrelated for the two promoters then we get

$$\rho(GFP, mCherry) = \frac{\eta^2(G)+\rho(L_{GFP},G)\eta(L_{GFP})\eta(G)+\rho(L_{mCherry},G)\eta(L_{mCherry})\eta(G)}{\eta(GFP)\,\eta(mCherry)} \qquad (29)$$

where we have written L_{GFP} and $L_{mCherry}$ for capacities of the pathways that lead to the activation of the GFP and mCherry gene respectively. The uncorrelated part of the average η^2 will now include a contribution from the pathway variation; and the quantity *Z*(*gfp*, m*Cherry*) includes this extra contribution. We get

$$Z(GFP, mCherry) = \frac{\eta^2(P_{GFP})+\eta^2(P_{mCherry})}{2} + \frac{\eta^2(\gamma_{GFP})+\eta^2(\gamma_{mCherry})}{2} \qquad (30)$$

where $\eta^2(\lambda_{GFP})$ is the transmission noise for the pathway that leads to GFP activation and $\eta^2(P_{GFP}) = \eta^2(L_{GFP}) + \eta^2(\lambda_{GFP})$, and similarly for $\eta^2(\lambda_{mCherry})$ and $\eta^2(P_{mCherry})$.

Thus, when uncorrelated expression variation for two independent promoters includes an average variation in pathways activation and gene expression variations for each promoter. The latter can be calculated from type I experiments. Knowing expression variations $\eta^2(\gamma)$ for each promoter and uncorrelated variation *Z* from equation (23) allows to calculate of pathway variations $\eta^2(P)$. To split contribution of variation of each pathway we need to perform three experiments with pairwise analysis of three promoters and then solve a system linear equations to calculate $\eta^2(P)$ of each pathway.

Below we give an example with expression of three promoters, vit-2, eft-3, hsp-90, in cells int3V and int3D.

$$Z\left(P_{eft-3} :: BFP, P_{vit-2} :: mCherry\right) = \frac{\eta^2(P_{eft-3})+\eta^2(P_{vit-2})}{2} + \frac{\eta^2\left(\gamma_{P_{eft-3}::BFP}\right)+\eta^2\left(\gamma_{P_{vit-2}::mCherry}\right)}{2} \quad (31)$$

$$Z\left(P_{eft-3} :: BFP, P_{hsp-90} :: mCherry\right) = \frac{\eta^2(P_{eft-3})+\eta^2(P_{hsp-90})}{2} + \frac{\eta^2\left(\gamma_{P_{eft-3}::BFP}\right)+\eta^2\left(\gamma_{P_{hsp-90}::mCherry}\right)}{2} \quad (32)$$

$$Z\left(P_{hsp-90} :: GFP, P_{vit-2} :: mCherry\right) = \frac{\eta^2(P_{hsp-90})+\eta^2(P_{vit-2})}{2} + \frac{\eta^2\left(\gamma_{P_{hsp-90}::GFP}\right)+\eta^2\left(\gamma_{P_{vit-2}::mCherry}\right)}{2} \quad (33)$$

Average gene expression noise of two promoters, e.g. $\frac{\eta^2\left(\gamma_{P_{eft-3}::BFP}\right)+\eta^2\left(\gamma_{P_{vit-2}::mCherry}\right)}{2}$ is calculated from type I experiments and equation (28). It is therefore a known value for each cell type and promoter. We calculate quantity *Z* directly from equation (23). Quantity *Z* and average expression noise therefore give a particular number of each of the equations above. We can rewrite them in more suitable form to calculate pathways variations

$$\frac{\eta^2(P_{eft-3})+\eta^2(P_{vit-2})}{2} = A_1 \quad (34)$$

$$\frac{\eta^2(P_{eft-3})+\eta^2(P_{hsp-90})}{2} = A_2 \quad (35)$$

$$\frac{\eta^2(P_{hsp-90})+\eta^2(P_{vit-2})}{2} = A_3 \quad (36)$$

Where A_1, A_2 and A_3 are known values. To calculate pathway variation for a particular promoter, e.g. *eft-3*, we will sum up (34) and (35) and subtract (36):

$$\frac{\eta^2(P_{eft-3})+\eta^2(P_{vit-2})}{2}+\frac{\eta^2(P_{eft-3})+\eta^2(P_{hsp-90})}{2}-\frac{\eta^2(P_{hsp-90})+\eta^2(P_{vit-2})}{2}=A_1+A_2-A_3 \quad (37)$$

$$\frac{\eta^2(P_{eft-3})+\eta^2(P_{vit-2})+\eta^2(P_{eft-3})+\eta^2(P_{hsp-90})-\eta^2(P_{hsp-90})-(P_{vit-2})}{2}=A_1+A_2-A_3 \quad (38)$$

$$\frac{2\eta^2(P_{eft-3})}{2}=A_1+A_2-A_3 \quad (39)$$

$$\eta^2(P_{eft-3})=A_1+A_2-A_3 \quad (40)$$

Thus, we have got one estimate of $\eta^2(P_{eft-3})$. To obtain it we used results of the experiments involving P_{eft-3}, P_{vit-2} and P_{hsp-90} reporters in pairwise manner. We say that these 3 pairs of experiments form a 'triangle'. However, we could choose another triangle consisting of experiments with P_{eft-3}, P_{vit-2} and $P_{hsp-16.2}$ reporters, or a triangle of P_{eft-3}, $P_{hsp-16.2}$ and P_{hsp-90} reporters. To get a better estimate of the true $\eta^2(P_{eft-3})$ value we have calculated it from all three possible triangles and averaged. We did the same for other reporters too. The resulting $\eta^2(P)$ values are shown in boxplots in Figure 3.

Similarly, we can calculate pathway variation for other promoters for which we have measured their expression noise. In cases when we did not measure expression noise, e.g. *hsp-17* promoter, we did calculate pathway variation for these promoters as well. In these cases we have limited our analysis with comparison of total correlated variation of two promoters to their uncorrelated variation. In all cases we have observed that correlated variation dominates over uncorrelated term:

$$\frac{Cov(P_{hsp-17}::GFP, P_{eft-3}::mNeptune)}{\overline{P_{hsp-17}::GFP} \times \overline{P_{eft-3}::mNeptune}} \gg Z(P_{hsp-17}::GFP, P_{eft-3}::mNeptune)$$

Supplementary Note 6: Additional Correlations between Phenotypes and Reporter Genes

As in previous reports, we also find activities of gene products correlate with the expression levels of fluorescent reporter genes at other points in life. Previous reports showed the *hsp-16.2* and *hsp-90* chaperone biomarkers covaried with the activities of mutant proteins, discerned from the mutant phenotypes that did or did not manifest during larval development. We used the *hsp-16.2* biomarker gene to sort adult animals expressing an incompletely penetrant, dominant Ras gain of function mutation, which causes animals to develop between zero and four hypodermal neoplasias. If animals that had higher Ras activity had higher gene expression capacity that persisted into adulthood, we would expect the *hsp-16.2* biomarker to correlate with the penetrance and expressivity of the Ras mutation. Supplementary Fig. 13 shows that animals that make less of the biomarker have a lower penetrance; brighter animals have more hypodermal neoplasias. This evidence supports the other evidence showing that animals vary in effective gene dosage for multiple different genes simultaneously. Supplementary Fig. 14 shows images of animals in which the nuclear envelope of the somatic cells, including the neoplastic hypodermal cells, is delineated by a green fluorescent protein fusion to Emerin, a nuclear envelope component. Note that, even without seeing the covariation with other genes, the individual animals with distinct numbers of growths emanating from their ventral surface provides obvious evidence of differences in Ras activity between animals. We are only showing evidence of previous Ras activity is covarying with chaperones after adult heat shock. The relationship between elevated Ras and chaperone activity is not new or unknown; chaperone-targeting drugs have been used to suppress Ras activity in cancer treatment[1,2].

Next, we tested the hypothesis that some other, non-native larval gene activity could be predicted by expression of a reporter gene. We tested the hypothesis that we could detect correlations between a ubiquitously expressed reporter that correlated well with chaperone biomarkers, $P_{eft\text{-}3}$*::GFP*, and the activity of the neomycin resistance gene controlled by another promoter, *rps-27*. We sorted diapaused larval (L1) animals on $P_{eft\text{-}3}$*::GFP* expressed from a site on chromosome I; these animals expressed a neomycin resistance gene (NeoR) on a different chromosome (chromosome II) under control of the *rps-27* promoter. If general protein expression capacity (*G*) was a major mechanism of intestine cell-to-cell (and animal-to-animal) variation, then animals that have more GFP should have more NeoR and resist extreme neomycin concentrations to a greater extent.

Supplementary Fig. 15 shows that we found this to be the case; brighter green animals can grow to adulthood in a high neomycin environment better than dimmer animals.

The data from these experiments is consistent with prior observations of covariation between fluorescent reporters and penetrance of mutant phenotypes previously reported in larval worms [3,4]. Taken together with all of the previous data on covariation and the data on covariation between reporter genes in this manuscript, the case for chaperone biomarkers revealing physiological states of different proteome dosage (at least for some significant portions of the proteome) seems well supported. For this entire manuscript, the data comprising these covariation measurements consists of many distinctly regulated promoters, proteins and loci (see Methods), using four distinct fluorescent proteins, originating from the zooxanthellae endosymbionts of three different cnidarians. Thus, these results cannot be an artifact of some property of any single promoter, protein, locus or fluorescent protein (e.g., folding, maturation, quantum efficiency, bleaching, pKa).

Supplementary Figure 13. Penetrance and expressivity of the Ras gain of function mutation is correlated with *hsp-16.2* expression levels. Penetrance of Ras/*let-60* gain of function mutation *let-60(n1046)* correlates with the ability to express $P_{hsp\text{-}16.2}$*::GFP* reporter. Chi Square for distributions of bright or dim vs unselected $P < 0.0001$. Error bars show standard error. Difference in penetrance between bright and dim $P < 0.05$, Paired t-test from three independent experiments scoring 1,120 animals. See Supplementary Table 1 for raw numerical data.

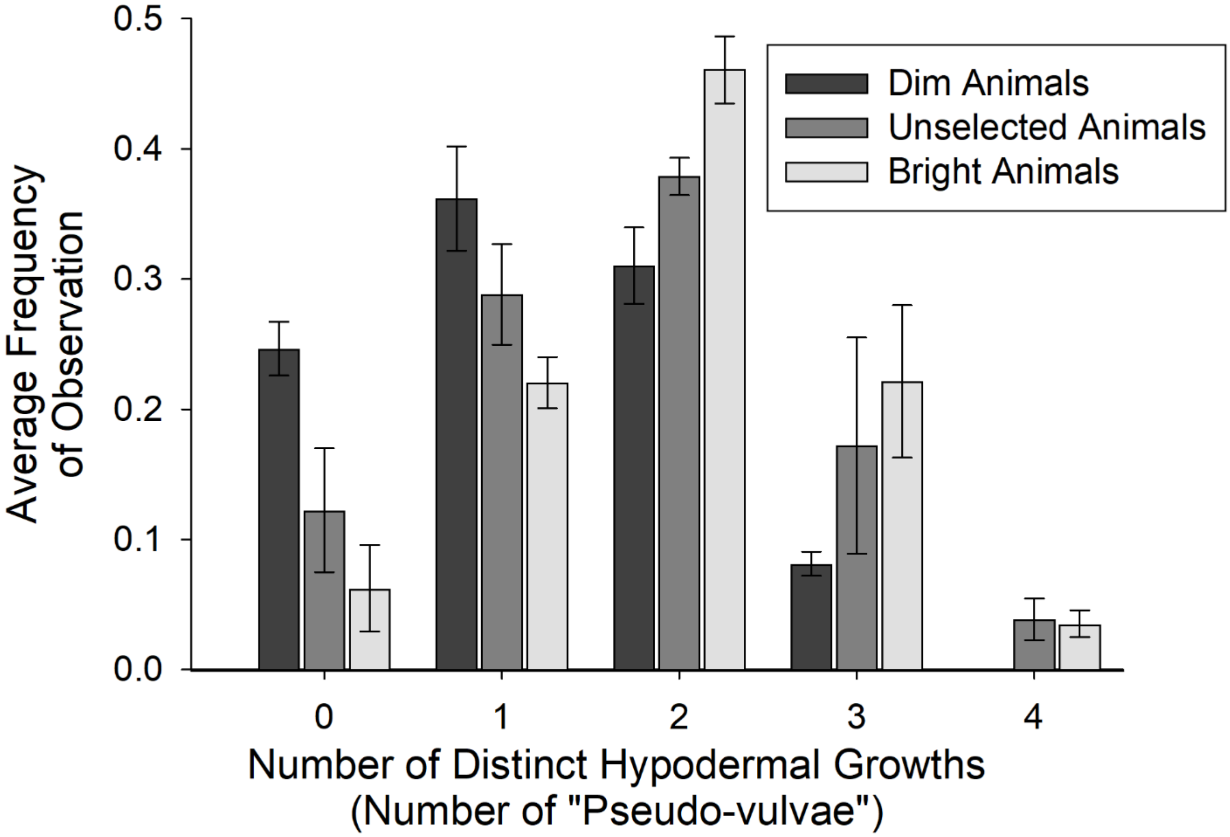

<u>Supplementary Figure 14. Penetrance and expressivity of Ras gain of function mutation in *C. elegans.*</u>

Animals express EMR-1::GFP to mark cell nuclei. Wild type and superficially wildtype *let-60(n1046)* animals are shown in the top two panels. White scale bar in bottom right of top panel is ten micrometers. Some *let-60(n1046)* mutants do not develop extra pseudo-vulvae/neoplasias (impenetrant, phenotypically wild type). Other *let-60(n1046)* animals exhibit variable numbers of hypodermal neoplasias. See methods for details of microscopy.

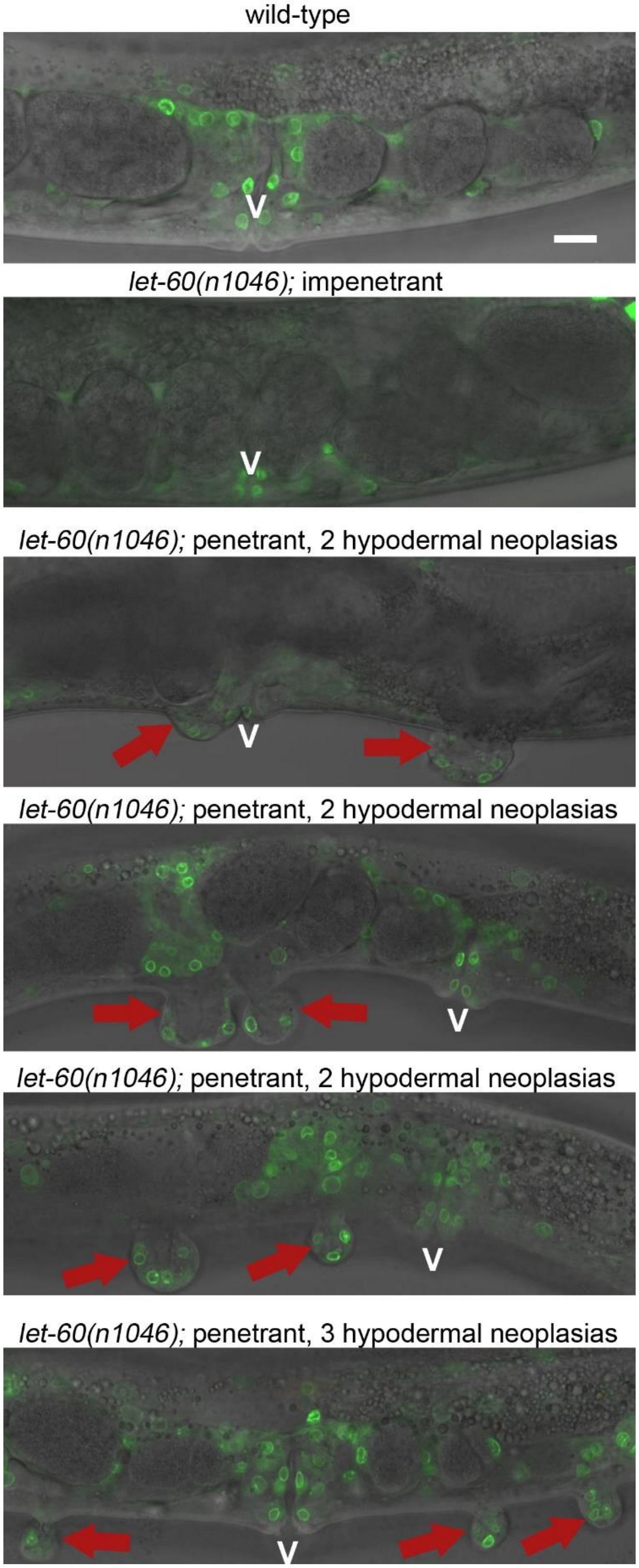

Supplementary Table 1: Number of Hypodermal Neoplasias Organized by Experiment.

Experiment 1

# Growths	Dim		Unselected		Bright	
	# Animals	% Animals	# Animals	% Animals	# Animals	% Animals
0	10	23%	2	3%	0	0%
1	19	44%	15	21%	7	19%
2	11	26%	25	35%	15	42%
3	3	7%	24	34%	12	33%
4	0	0%	5	7%	2	6%
Total # Animals	43		71		36	

Experiment 2

# Growths	Dim		Unselected		Bright	
	# Animals	% Animals	# Animals	% Animals	# Animals	% Animals
0	27	29%	31	16%	6	7%
1	30	32%	66	34%	21	26%
2	30	32%	79	40%	41	51%
3	7	7%	16	8%	11	14%
4	0	0%	5	3%	2	2%
Total # Animals	94		197		81	

Experiment 3

# Growths	Dim		Unselected		Bright	
	# Animals	% Animals	# Animals	% Animals	# Animals	% Animals
0	42	22%	45	18%	18	11%
1	62	32%	79	32%	33	21%
2	68	36%	95	38%	73	46%
3	19	10%	24	10%	31	19%
4	0	0%	5	2%	4	3%
Total # Animals	191		248		159	

Supplementary Figure 15. Animals expressing the NeoR gene that express more *Peft-3::mEGFP* in the L1 Diapause Develop to Adulthood Better on Neomycin. Drug resistance conferred by Neo^R expressed from $P_{rps\text{-}27}$ promoter correlates with expression of $P_{eft\text{-}3}$*::EGFP* reporter. $P_{eft\text{-}3}$*::EGFP* reporter does not predict ability to grow in the absence of neomycin (striped bars – from two independent experiments for bright and dim scoring 171 animals, and three independent experiments for unselected animals scoring 138 animals). High $P_{eft\text{-}3}$*::EGFP* expression predicts a significant difference in the ability to grow to adulthood on a high neomycin concentration (solid bars; $p < 0.05$, Paired t-test from five independent experiments scoring 443 animals). Error bars show standard error. See Supplementary Tables 2 and 3 for raw numerical data.

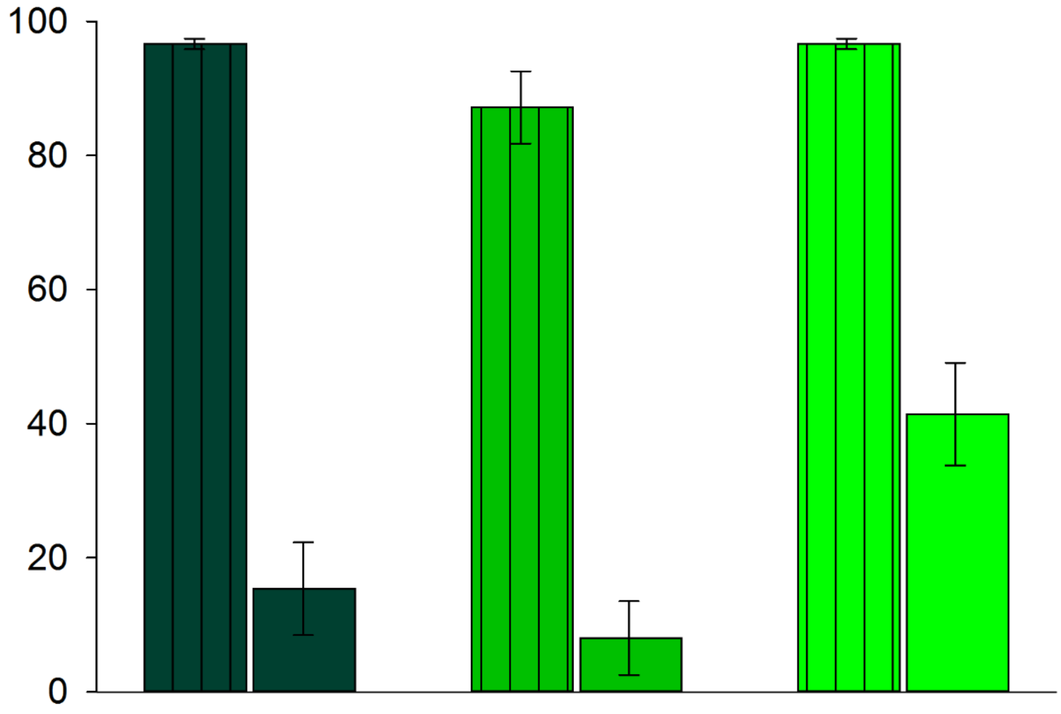

Supplementary Table 2. Growth to gravid adulthood after 72 hours development on regular NGM.

Experiment	Bright adulthood	Bright total	Bright % adulthood	Dim adulthood	Dim total	Dim % adulthood	Unselected adulthood	Unselected total	Unselected % adulthood
1							37	45	82%
2									
3	46	48	96%	46	48	96%	48	49	98%
4									
5	38	39	97%	38	39	97%	36	44	82%

Supplementary Table 3. Growth to gravid adulthood after 96 hours on high Neomycin concentration NGM.

Experiment	Bright neo adulthood	Bright neo total	Bright neo % adulthood	Dim neo adulthood	Dim neo total	Dim neo % adulthood	Unselected neo adulthood	Unselected neo total	Unselected neo % adulthood
1	15	48	31%	2	43	5%	1	45	2%
2	26	46	57%	1	42	2%			
3	25	49	51%	15	51	29%	9	47	19%
4	25	48	52%	15	43	35%			
5	6	37	16%	2	36	6%	1	40	3%

Supplementary Note 7: Persistence of Physiological States.

Physiological states are not always persistent. Our previous reports showed that the high gene expression capacity state revealed by the *hsp-16.2* biomarkers was persistent enough to mean a difference in lifespan[5,6], and also heritable[7]. In the scenario above wherein the animals had a Ras gain of function mutation, animals that developed more neoplasias during L3/L4 larval development expressed more of the *hsp-16.2* biomarker after an adult heat shock. Thus, the state of high Ras expression, or the consequence, was persistent enough to influence adult physiology. Some states may not manifest without some stress and some states may not persist.

We quantified protein expression capacity in embryos and L1 larvae (the time point we measured *eft-3*), and found that the expression level of the *vit-2::GFP* knockin in embryos or $P_{eft\text{-}3}$*::GFP* in L1 larvae, respectively, did not correlate with adult gene expression capacity. See Supplementary Fig. 16 showing the correlation of L1s and embryos with adult expression levels.

We attempted to determine persistence of other larval states with the adult states. However, we were confounded by current technical and biological limitations. Specifically, we were unable to reliably determine persistence of other larval states (correlation with adult states) because our current anesthesia-based mounting technology confounds our results; we get opposing results with or without anesthesia. And, our no-anesthesia results are currently too technically noisy to rely upon for longitudinal measures with adults for anything other than embryos or L1s.

In our preliminary observations, we were able to determine some L2-L4 larvae are developmentally out of phase, made apparent by the differences in expression bursting (indicated by only seeing deviation towards the faster-maturing mCherry). The in-phase larvae had differences in gene expression capacity. How much differences in *G* or being out of phase (maybe construed as a signaling or timing difference) contribute to subsequent outcomes and establishment of persistent or transient states remains undetermined. So, to reiterate, we observed some animals were at steady state gene expression levels for a given larval phase, and others are still bursting to get into steady larval state; this cell-autonomous bursting was revealed by differential maturation of reporter proteins (animals only deviated in the faster maturing mCherry direction, Supplementary Fig. 17).

In our experiments, the early embryonic/L1 state did not persist, but was consequential (NeoR). The lack of persistence of the embryonic or early L1 state may be due to the fact that embryos can develop to the L1 state without any ribosomes in their genome[8] – suggesting that there is a switch between maternal ribosomes and self-made ribosomes when the animals hatch and exit the L1 diapause and begin feeding. However, the Lehner group's reports support the idea of persistence of physiological states in two ways. The first evidence suggests persistence of physiological states because chaperone reporters could predict subsequent developmental events[3,4]. The second evidence is that differences in the response to larval heat shock persisted into adulthood, affecting ovulation rates[3]. Additionally, we did detect evidence of persistence or some relationship between the larval and adult states when animals bore a Ras gain of function mutation.

Supplementary Figure 16. High protein expression capacity in the embryonic or L1 diapause states does not persist into adulthood.

a. Average intensity of Peft-3::GFP in the same animals at L1 larval stage and on Day 2 of adulthood is shown. Two panels represent independent experiments. For imaging, animals were mounted on 1% agarose pads and covered by a cover slip. No anesthesia was used for L1 larvae; adult animals were anesthetized with 0.2%Tricaine/0.02% Tetramisole. L1 larvae were singled onto individual NGM plates after imaging. Panel **b** shows yolk content in 2-cell stage embryos (visualized through knock-in GFP tagged Vit-2 protein) and adult expression of *hsp-90* reporter in the same animals on Day 2 of adulthood. Three panels represent three biological replicates. For imaging, adult animals were anesthetized with 0.2%Tricaine/0.02% Tetramisole, mounted on 1% agarose pads and covered by a cover slip. Eggs were imaged without anesthesia and then singled onto individual NGM plates. Exact numbers of animals in each experiment are indicated by the number of dots on each scatter plot.

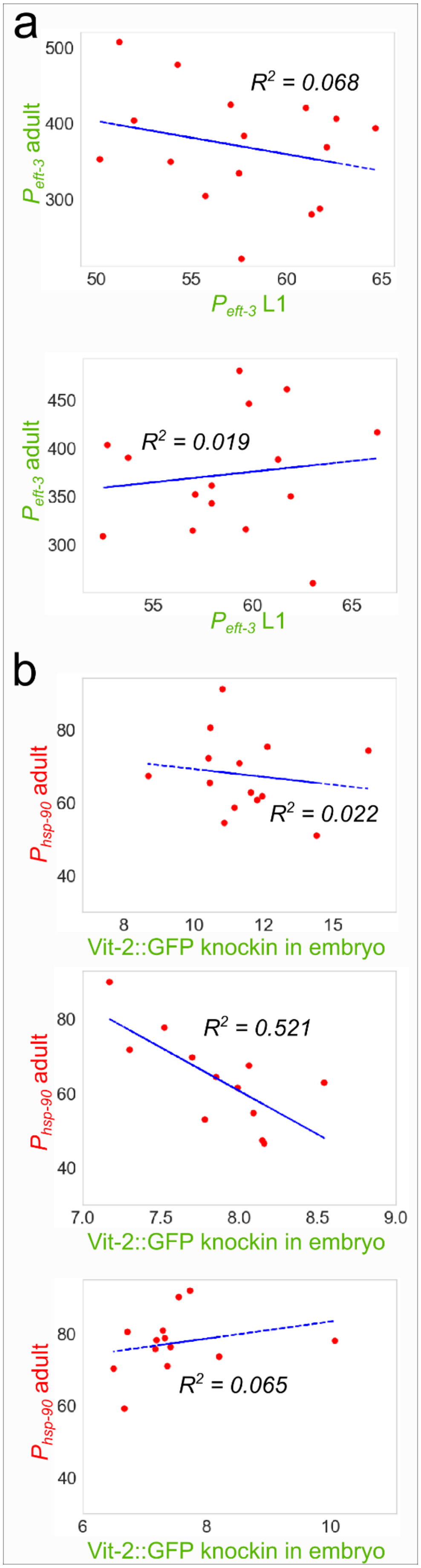

Supplementary Figure 17. Larval stages exhibit out-of-phase developmental asynchrony punctuated by cell autonomous protein bursting followed by a period of developmentally in phase differences in protein expression capacity. The left panel of the figure below shows Type I measurements made in young L4 animals where we saw deviation only in the faster maturing mCherry protein, suggesting the animals were not yet in steady state (compare to perfectly yellow animal on the right panel); white scale bars in bottom right of images are 50 micrometers. If we look at animals a few hours later, we see that animals are mostly expressing the same amounts of both alleles, with equivalent minor deviations towards both the mCherry and the mEGFP allele (right panel). We saw the same thing with Type II experiments in L2 and L3 animals; deviation only in the mCherry direction in some cells in some animals, followed by a period of seemingly steady state expression wherein the animals varied mostly in the *G* component. It is currently unclear how much this developmental asynchrony and cell autonomous protein bursting before entry into steady state contribute to differences in biological outcomes. Hence, while our microscopic approach is suitable to investigate gene expression in adults, the same type of analysis in rapidly developing larvae is currently confounded by rapidly changing developmental landscape and bursts of protein synthesis. More technical work will need to be done to quantify these developmental cell autonomous bursting events to determine and their underlying causes and consequences. Specifically, they could be caused by signaling perception differences or expression capacity differences, and they may or may not contribute to or result from physiological states of high or low protein expression capacity or different developmental trajectories. To make these determinations we performed three independent experiments measuring expression in L2 and L4 animals with ten animals per group. We also performed the same number of experiments with L3 animals, measuring ten animals per experiment. In each case, we saw in phase and out of phase animals, but we did not observe variation in the green channel, indicating that we did not detect any true signaling noise differences, just autonomous bursts of protein expression in individual cells.

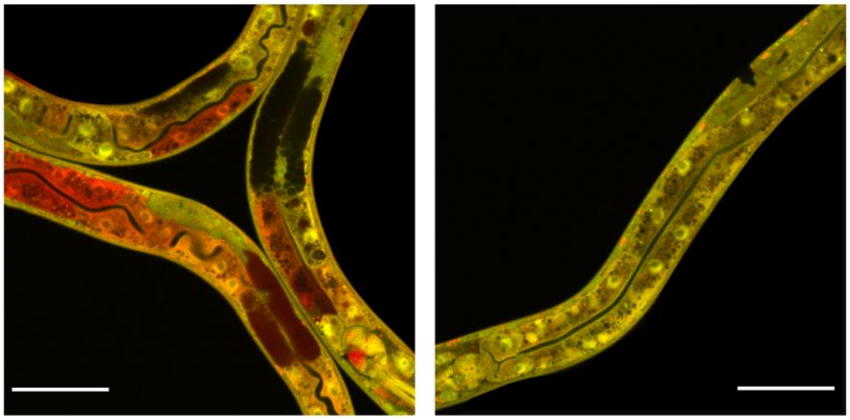

Supplementary Note 8: Trade-offs

Animals Can Make Trade-Offs, But They May Not Always Do So. Previous reports showed evidence about trade-offs between stress resistance or lifespan, and progeny production mediated by insulin signaling. Two prior reports showed that the insulin signaling system increased lifespan at cost to phenotypic plasticity and progeny production[9,10]. Another report showed that animals that respond better to heat shock as larvae, in terms of lower insulin signaling, have a lowered ovulation rate as adults[3]. So, better response to stress may come at a cost to progeny production. And, we know that the insulin signaling systems also regulates biomarker variation[11] and levels[12], in addition to mediating the adult trade-off between stress resistance/lifespan and progeny production[3]. On the contrary, heat shocks to adults did not reveal trade-offs between animals that responded better or worse (as indicated by the *hsp-16.2* biomarker)[7]. Additionally, no trade-off between self-fertility and lifespan has been observed in unperturbed *C. elegans*[13,14]. One of our working models suggests that animals that express more biomarker do so at the cost of a trade-off with progeny production. Alternatively, animals may express more biomarker simply because the animals that express less are frailer overall. Yet, the dimmer animals did not produce fewer progeny, despite living less time[7]; however, it is unclear if poor responders to heat shock ovulated at the same rate – if they produced more progeny per unit time.

We decided to use an experimental hammer to determine if the insulin signaling system could mediate a trade-off after another distinct, but chaperone-related, adult stress. We used genetic models of high and low insulin signaling (*daf-16* and *daf-2,* respectively), and genetic models of lowered nutrient sensing (*nhr-49*) and compromised heat shock response (*hsf-1*); alleles listed in materials and methods. We irradiated wild-type and mutant one day old adult animals with 1000 Joules of UV radiation and measured subsequent lifespan and progeny production. Under these conditions, across genotypes, we saw a tradeoff between progeny production and stress resistance that is mediated by insulin signaling, shown in Supplementary Fig. 18. The figure also shows genetic evidence (the *hsf-1* mutant faring poorly) that the heat shock response was also required for somatic and germline survival; even the seemingly malnourished, sickly *nhr-49* animals did better. Hence, which we were able to observe a trade-off after damaging irradiation with mutants, but not after heat shock within a population of high and low responders.

This data supports a general bet-hedging model wherein insulin signaling mediates interindividual differences in stress perception and/or response resulting trade-offs between somatic maintenance and progeny production among members of isogenic populations, ensuring the fitness of some members of the population in a variety of scenarios. However, it does not negate the fact that we did not detect a trade-off in heat shocked adults in our prior report. It may be that trade-offs cannot be made in wild-type animals after an adult heat shock; that is the notion most supported by the data.

Supplementary Figure 18. Trade-offs between lifespan and progeny production among different mutants.

Panel **a** shows a figure legend. Panels **b-d** show lifespan after irradiation by 1000 Joules of UV radiation for different *C. elegans* strains. Panels **e**&**f** show boxplots of fecundity for five individual animals for each genotype/condition, with Panel **f** showing the full dynamic range, including non-irradiated wild-type animals. The boundary of the box closest to zero indicates the 25th percentile, a line within the box marks the median, a dash within the box marks the average, and the boundary of the box farthest from zero indicates the 75th percentile. Whiskers above and below the box indicate the 90th and 10th percentiles. We measured lifespan for 50 animals in three independent experiments for each genotype. We measured the self-fertile fecundity of five irradiated individual animals from each genotype in five independent experiments, along with a non-irradiated wild-type control (30 total animals from five independent experiments).

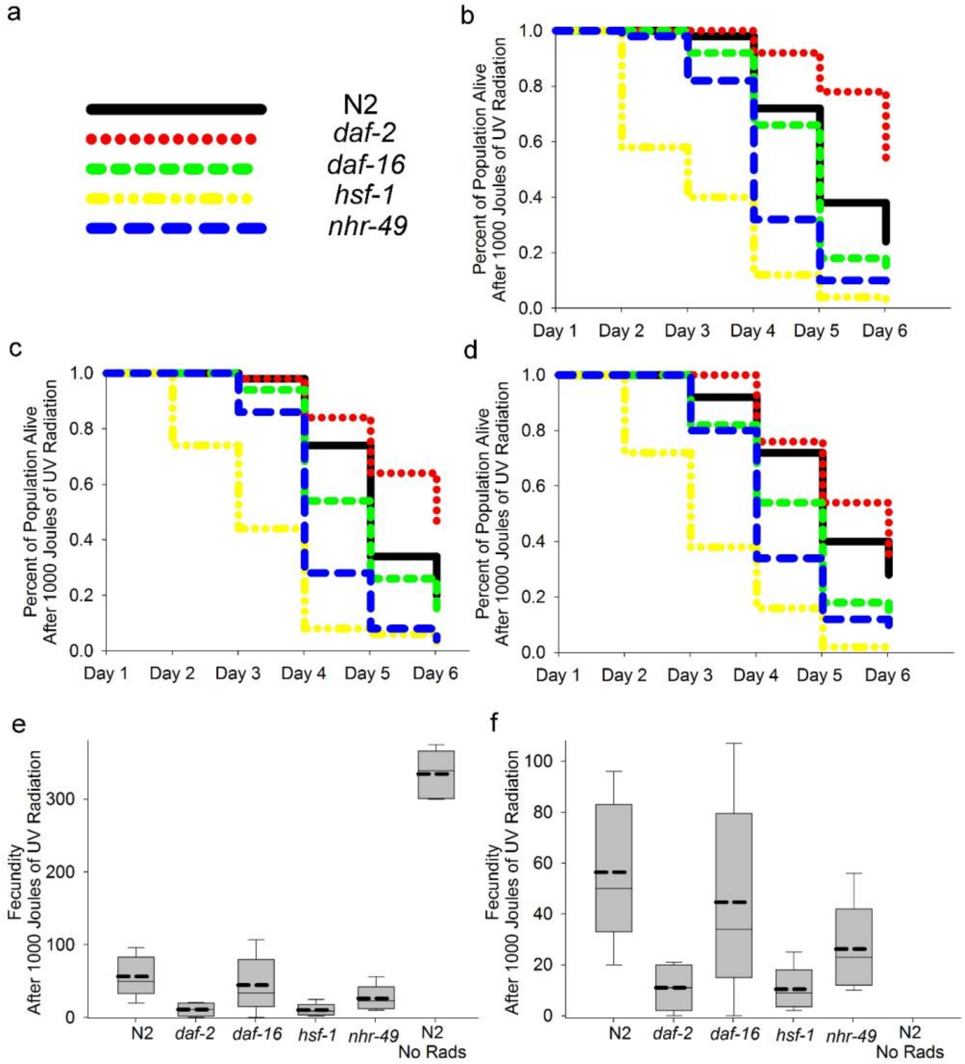

Supplementary Table 4

Supplementary Table 4: Crosses for Type I and Type II Experiments
P_{daf-21}::*gfp* (RBW2661) x P_{daf-21}::*mcherry* (RBW2642)
P_{daf-21}::*gfp* (RBW2661) x P_{vit-2}::*mcherry* (RBW2581)
$P_{hsp-16.2}$::*gfp* (RBW2601) x P_{daf-21}::*mcherry* (RBW2642)
$P_{hsp-16.2}$::*gfp* (RBW2601) x $P_{hsp-16.2}$::*mcherry* (RBW2561)
$P_{hsp-16.2}$::*gfp* (RBW2601) x P_{vit-2}::*mcherry* (RBW2581)
P_{vit-2}::*gfp* (RBW2621) x P_{vit-2}::*mcherry* (RBW2581)
P_{mtl-2}::*mcherry* (RBW2531) x P_{daf-21}::*gfp* (RBW2661)
P_{mtl-2}::*mcherry* (RBW2531) x $P_{hsp-16.2}$::*gfp* (RBW2601)
P_{mtl-2}::*mcherry* (RBW2531) x P_{vit-2}::*gfp* (RBW2621)
P_{eft-3}::*mtagbfp2* (ARM6) x P_{daf-21}::*mcherry* (RBW2642)
P_{eft-3}::*mtagbfp2* (ARM6) x $P_{hsp-16.2}$::*mcherry* (RBW2561)
P_{eft-3}::*mtagbfp2* (ARM6) x P_{mtl-2}::*mcherry* (RBW2531)
P_{eft-3}::*mtagbfp2* (ARM6) x P_{mtl-2}::*mcherry* (RBW2531)
P_{eft-3}::*mtagbfp2* (ARM6) x P_{eft-3}::*mneptune* (ARM5)
P_{hsp-17}::*gfp* (RBW3211) x P_{daf-21}::*mcherry* (RBW2642)
P_{hsp-17}::*gfp* (RBW3211) x $P_{hsp-16.2}$::*mcherry* (RBW2561)
P_{hsp-17}::*gfp* (RBW3211) x P_{mtl-2}::*mcherry* (RBW2531)
P_{hsp-17}::*gfp* (RBW3211) x P_{vit-2}::*mcherry* (RBW2581)
P_{hsp-17}::*gfp* (RBW3211) x P_{eft-3}::*mneptune* (ARM5)
$P_{hsp-16.2}$::*mcherry* (RBW2561) x *emr-1::gfp* (stable RBW2)

Supplementary Discussion

In distinct alternative interpretations of the working model, there may, or may not, be trade-offs between somatic fitness and reproductive fitness, and, these trade-offs can manifest distinctly in terms of how they affect protein production or export from the intestine. In the first model interpretation, high chaperone/*hsp-16.2* biomarker expressing animals respond better to heat shock by making and maintaining protein better, they live longer, and there is no trade-off with progeny production, there are just winners and losers. This is most supported by the data.

It will be interesting in the future to determine these molecular mechanisms underlying the states of high and low global protein dosage. In another possible model, there is a trade-off, both bright and dim animals produce and maintain similar amounts of protein, but the dimmer animals simply export more protein to their oocytes, effectively atrophying the intestine faster as in[15]. In another possible trade-off model, the dimmer animals do make more protein to make more progeny, consistent with a trade-off after heat shock[3], and the brighter animals have more protein because of increased maintenance, and not better production. It is interesting to notice in this regard, that we have previously found that animals expressing high levels of *hsp-16.2* biomarker have lower levels of *mac-1*/*rix7p* ribosome export factor[16]. It is therefore intriguing to speculate that bright animals synthesize less protein and simply maintain mature proteins better. Consistent with this speculation, decreased insulin signaling is associated with decreased global protein turnover[17-19]. Yet, high biomarker expression resulting from decreased protein turnover is also not supported by our current results, as animals that made more timer protein under *eft-3* promoter control had the same ratio of relatively younger versus relatively older protein (Supplementary Fig. 12). Moreover, these alternative trade-off models are not supported by our current data[7]. Supplementary Note 8: Trade-offs and Supplementary Fig. 18 shows that we can detect trade-offs between lifespan and progeny production in other stressful biological scenarios, such as damaging UV radiation treatment. However, we do not detect such trade-offs after adult heat shock[7].

What might be happening with intestinal protein expression capacity at other times during development and aging? Here, we examined sources of variation in gene expression in the intestine cells of two-day old adult *C. elegans* that had or had not been heat shocked. For the non-heat shocked animals, this was the peak of reproductive output[20]. For the heat shocked animals, this is the point at which resistance to lethal thermal stress

and lifespan are predicted by *hsp-16.2* reporter levels. Our results show that intrinsic noise and signaling noise are relatively low, and that the major contribution to differences in gene expression is differences in general protein expression capacity/effective protein dosage. This axis of variation is dominant, whether animals are heat shocked or not. It is dominant in large or small populations of animals. Observations from reporter proteins, fusion proteins and phenotype are all consistent with the idea that expression of significant portions of the proteome covaries with expression of chaperones. While we did detect several instances of significant cell autonomous allele bias and signaling noise, these sources of variation did not dominate.

We found that differences in protein expression capacity can be consequential in early larval development, but do not appear to persist into adulthood (at least not from the L1 diapause), consistent with a prior report showing embryonic physiology (yolk protein amount) did not correlate with adult physiology (lifespan)[21]. We looked at larval animals with and without heat shock and found cell autonomous bursts of protein expression and variation in gene expression capacity, which will require further investigation, as we do not know if the cells burst because of differences in signal reception or because of some other autonomous difference. In adults, we found that high *G* animals maintain significantly higher fractions of both old and young protein than low *G* animals, with or without heat shock. Thus, the animals responding better to heat shock are better able to produce and maintain protein in their intestines. Given the recent finding that the intestine consumes itself[15], the 2011 study wherein Sanchez-Blanco et al showed that most reporter genes they measured in the intestine predicted lifespan seems more logical[22]; more remaining protein to burn equated to more lifespan.

It is worth noting that some of the phenotypes predicted by or correlated with chaperone reporters have to do with the action of genes in the polyploid intestine and hypodermis (*tbx-9*, *lin-31* and *vab-9* are expressed in the hypodermis[3,4]). This is also true for the Ras gain of function affecting vulva development, reported here, and at least partially true for the more organismic phenotypes of thermotolerance, lifespan and neomycin resistance, which must rely on the intestine and hypodermis, at least in part. We mention this again because polyploidy cells may buffer against other kinds of variation, like intrinsic noise[23], and may be responsible for the dominance of the *G* axis, at least in adult intestines, and probably in the polyploidy hypodermis. While we do not know exactly what kind of variation is happening in larval cells, it is worth reconsidering some of the results from Casanueava et al. on penetrance. In 2012, Casanueva et al. showed that overexpression of HSF-1, which

activates expression of chaperones, decreased penetrance of hypomorphic mutations, but not of null mutations. The mutations we believe to be null were listed as "non-TS/Cold-sensitive"[10]; we believe they are almost certainly null because these alleles (*lin-31(gk569) & efn-2 (ev568)*) delete most of the exons, leaving only the final exon for each gene (www.wormbase.org).

How could chaperones influence the dosages of other proteins in intestine cells? Given the role that chaperones play in protein production, maintenance and turnover, it is not surprising that chaperone reporters predict differences in biological outcomes, from penetrance of mutations to lifespan[24]. What was initially surprising was the covariation of expression of these reporters with other distinctly regulated genes. This fact can be explained if expression of these genes is affected by differences in protein expression capacity. Though, again, in retrospect, given the role of chaperones, it should not have been surprising. And, given that metazoans have to coordinate the activities of multiple cell types in multiple tissue types, it may not be surprising that signaling noise was incredibly restricted, relative to the one other single celled eukaryote in which these kinds of measurements have been made (brewer's yeast).

Another source of variation in protein dosage could be variation in genome dosage. Indeed, our previous work showed that animals that were heterozygous for the biomarker expressed about half as much reporter protein[25] – so, like other systems, gene dosage affects expression level in *C. elegans*. However, due to technical limitations of our fixation procedure, we were not able to reliably make simultaneous measurements of fluorescent protein and nuclear genome content to determine any correlation between ploidy and gene expression level. We did not pursue this further because, currently, we do not think variation in intestinal ploidy drives variation in intestinal proteome dosage for two reasons. First, we see that the variation in biomarker expression is controlled by AFD neuron depolarization[11], which has no known role in ploidy control, nor does the *gcy-8* mutant we used to shut down AFD depolarization have the ploidy-related smaller body phenotype[26]. Second, in other work focused on cis control of intrinsic noise, we found that diploid muscle cells have about the same amount of extrinsic noise (revealed here as *G*) as polyploid intestine cells[23].

The high correlation of chaperone reporters with phenotypes (Supplementary Figs. 13-15 and [3,5,6,27]), distinctly regulated transcriptional reporters (Fig. 3), fusion proteins (Fig. 4) and knockins (Fig. 4) suggests that

at least some significant portion of the cellular proteome covaries with chaperone biomarkers in some circumstances. This is worth considering in the larger context of any biological scenario. However, we do not yet know what fraction of the cellular proteome for which protein dosage correlates with chaperone abundance. Our experimental system did detect gene expression levels changing in response to external signals (Supplementary Fig. 7), and instances of clearly detectable cell autonomous intrinsic noise and signaling noise (for intrinsic noise see Supplementary Fig. 4 *hsp-90* ring four; for signaling noise see Supplementary Fig. 9 *hsp-16.2 & mtl-2* ring one, and *hsp-17 & vit-2* ring one). However, for the most part, the small fraction of the genome we examined covaried fairly well (e.g., Figs. 3&4). Presumably, additional work exploring variation in the expression or activity of the myriad of remaining genes will identify additional axes of variation that will help define specific fractions/modules of the proteome that covary in different scenarios. For example, Sanchez-Blanco et al found another, uncorrelated axis of variation using a fluorescent protein reporters[22]. While most reporters they looked at in eight day old adult worm intestines were fairly well-correlated (e.g, r = 0.4-0.87), one single reporter, controlled by the promoter for *C26B9.5*, which encodes a serine protease, did not correlate well[22]. While we did not observe major uncorrelated variation, it may be possible to find significant differences in P or γ, with additional fluorescent reporter genes or fluorescent knockins, or in other experimental scenarios. In fact, in another study we find that γ, intrinsic noise of allele expression, can be much greater[23].

Supplementary References

1 De Raedt, T. *et al.* Exploiting cancer cell vulnerabilities to develop a combination therapy for ras-driven tumors. *Cancer Cell* **20**, 400-413, doi:10.1016/j.ccr.2011.08.014 (2011).
2 Acquaviva, J. *et al.* Targeting KRAS-mutant non-small cell lung cancer with the Hsp90 inhibitor ganetespib. *Molecular cancer therapeutics* **11**, 2633-2643, doi:10.1158/1535-7163.MCT-12-0615 (2012).
3 Casanueva, M. O., Burga, A. & Lehner, B. Fitness trade-offs and environmentally induced mutation buffering in isogenic C. elegans. *Science* **335**, 82-85 (2011).
4 Burga, A., Casanueva, M. O. & Lehner, B. Predicting mutation outcome from early stochastic variation in genetic interaction partners. *Nature* **480**, 250-253 (2011).
5 Rea, S. L., Wu, D., Cypser, J. R., Vaupel, J. W. & Johnson, T. E. A stress-sensitive reporter predicts longevity in isogenic populations of Caenorhabditis elegans. *Nature genetics* **37**, 894-898 (2005).
6 Mendenhall, A. R. *et al.* Expression of a single-copy hsp-16.2 reporter predicts life span. *The journals of gerontology* **67**, 726-733 (2012).
7 Cypser, J. R. *et al.* Predicting longevity in C. elegans: fertility, mobility and gene expression. *Mechanisms of ageing and development* **134**, 291-297, doi:10.1016/j.mad.2013.02.003 (2013).
8 Cenik, E. S. *et al.* Maternal Ribosomes Are Sufficient for Tissue Diversification during Embryonic Development in C. elegans. *Developmental cell* **48**, 811-826 e816, doi:10.1016/j.devcel.2019.01.019 (2019).
9 Walker, D. W., McColl, G., Jenkins, N. L., Harris, J. & Lithgow, G. J. Evolution of lifespan in C. elegans. *Nature* **405**, 296-297 (2000).
10 Jenkins, N. L., McColl, G. & Lithgow, G. J. Fitness cost of extended lifespan in Caenorhabditis elegans. *Proceedings* **271**, 2523-2526 (2004).
11 Mendenhall, A., Crane, M. M., Tedesco, P. M., Johnson, T. E. & Brent, R. Caenorhabditis elegans Genes Affecting Interindividual Variation in Life-span Biomarker Gene Expression. *The journals of gerontology*, doi:10.1093/gerona/glw349 (2017).
12 Prahlad, V., Cornelius, T. & Morimoto, R. I. Regulation of the cellular heat shock response in Caenorhabditis elegans by thermosensory neurons. *Science* **320**, 811-814 (2008).
13 Huang, C., Xiong, C. & Kornfeld, K. Measurements of age-related changes of physiological processes that predict lifespan of Caenorhabditis elegans. *Proc Natl Acad Sci U S A* **101**, 8084-8089 (2004).
14 Wu, D., Tedesco, P. M., Phillips, P. C. & Johnson, T. E. Fertility/longevity trade-offs under limiting-male conditions in mating populations of Caenorhabditis elegans. *Experimental gerontology* **47**, 759-763, doi:10.1016/j.exger.2012.06.010 (2012).
15 Ezcurra, M. *et al.* C. elegans Eats Its Own Intestine to Make Yolk Leading to Multiple Senescent Pathologies. *Curr Biol*, doi:10.1016/j.cub.2018.06.035 (2018).
16 Gadal, O. *et al.* A nuclear AAA-type ATPase (Rix7p) is required for biogenesis and nuclear export of 60S ribosomal subunits. *The EMBO journal* **20**, 3695-3704, doi:10.1093/emboj/20.14.3695 (2001).
17 Dhondt, I. *et al.* FOXO/DAF-16 Activation Slows Down Turnover of the Majority of Proteins in C. elegans. *Cell reports* **16**, 3028-3040, doi:10.1016/j.celrep.2016.07.088 (2016).
18 Depuydt, G., Shanmugam, N., Rasulova, M., Dhondt, I. & Braeckman, B. P. Increased Protein Stability and Decreased Protein Turnover in the Caenorhabditis elegans Ins/IGF-1 daf-2 Mutant. *The journals of gerontology* **71**, 1553-1559, doi:10.1093/gerona/glv221 (2016).
19 Visscher, M. *et al.* Proteome-wide Changes in Protein Turnover Rates in C. elegans Models of Longevity and Age-Related Disease. *Cell reports* **16**, 3041-3051, doi:10.1016/j.celrep.2016.08.025 (2016).
20 McCarter, J., Bartlett, B., Dang, T. & Schedl, T. On the control of oocyte meiotic maturation and ovulation in Caenorhabditis elegans. *Developmental biology* **205**, 111-128 (1999).
21 Perez, M. F., Francesconi, M., Hidalgo-Carcedo, C. & Lehner, B. Maternal age generates phenotypic variation in Caenorhabditis elegans. *Nature* **552**, 106, doi:10.1038/nature25012

https://www.nature.com/articles/nature25012#supplementary-information (2017).
22 Sanchez-Blanco, A. & Kim, S. K. Variable pathogenicity determines individual lifespan in Caenorhabditis elegans. *PLoS genetics* **7**, e1002047 (2011).

23 Sands, B., Yun, S. R. & Mendenhall, A. Introns Control Stochasticity in Metazoan Gene Expression. *BioRxiv* **1**, 15 Preprint available at https://www.biorxiv.org/content/10.1101/746263v4 (2019).
24 Taipale, M., Jarosz, D. F. & Lindquist, S. HSP90 at the hub of protein homeostasis: emerging mechanistic insights. *Nature reviews. Molecular cell biology* **11**, 515-528, doi:10.1038/nrm2918 (2010).
25 Mendenhall, A. R., Tedesco, P. M., Sands, B., Johnson, T. E. & Brent, R. Single Cell Quantification of Reporter Gene Expression in Live Adult Caenorhabditis elegans Reveals Reproducible Cell-Specific Expression Patterns and Underlying Biological Variation. *PloS one* **10**, e0124289, doi:10.1371/journal.pone.0124289 (2015).
26 Lozano, E., Saez, A. G., Flemming, A. J., Cunha, A. & Leroi, A. M. Regulation of growth by ploidy in Caenorhabditis elegans. *Curr Biol* **16**, 493-498, doi:10.1016/j.cub.2006.01.048 (2006).
27 Yang, J. & Tower, J. Expression of hsp22 and hsp70 transgenes is partially predictive of drosophila survival under normal and stress conditions. *The journals of gerontology* **64**, 828-838 (2009).
